# Establishing a direct interaction between the 19,20-EDP analog SA-22 and SIRT3: impact on cardiac mitochondrial homeostasis

**DOI:** 10.3389/fphar.2026.1805965

**Published:** 2026-06-02

**Authors:** Joshua W. Kranrod, Robert Valencia, Mobina Heidari, Kamilia M. Ibrahim, Adeniyi Michael Adebesin, Sailu Munnuri, John R. Falck, John M. Seubert

**Affiliations:** 1 Faculty of Pharmacy and Pharmaceutical Sciences, University of Alberta, Edmonton, AB, Canada; 2 Cardiovascular Research Institute, University of Alberta, Edmonton, AB, Canada; 3 Department of Pharmacology, Faculty of Medicine and Dentistry, University of Alberta, Edmonton, AB, Canada; 4 Division of Chemistry, Departments of Biochemistry and Pharmacology, University of Texas Southwestern Medical Center, Dallas, TX, United States; 5 TCG GreenChem, Inc. Process R&D Center at Princeton South, Ewing, NJ, United States

**Keywords:** 19,20-EDP, analog, cardiac, epoxylipids, hypoxia-reoxygenation, mitochondria, mitophagy, oxylipins

## Abstract

**Background:**

Despite extensive study, the structural, metabolic, and mechanistic heterogeneity amongst polyunsaturated fatty acids (PUFA) have confounded identification of their molecular targets and roles in cardiovascular diseases. Previously our group demonstrated that the cardioprotective properties of both 19,20-epoxydocosapentaenoic acid (EDP), a CYP450-derived metabolite of docosahexaenoic acid (DHA), and a synthetic structural analog SA-22, were SIRT3-dependent. Thus, we explored the impact of this signaling on mitochondrial homeostasis in the context of hypoxic myocardial injury. SA-22 ligand binding was confirmed via SYPRO Orange thermal shift assay.

**Methodology:**

SIRT3 catalytic activity was measured using an acetylated HDAC fluorogenic substrate assay. Point mutagenesis experiments confirmed the involvement of residue SER149. H9c2 cells were used as an *in vitro* model of hypoxia/reoxygenation (HR) injury. Cells were deprived of oxygen for 24 h followed by a 6-h reoxygenation period wherein cells were treated with either vehicle, 19,20-EDP (1 µM), or SA-22 (1 µM), either with the pan-sirtuin inhibitor nicotinamide (NAM) (30 µM), or the SIRT3-selective inhibitor 3-(1H-1,2,3-triazol-4-yl)-pyridine (3-TYP) (50 µM). Mitophagy was assessed via the pH-dependent fluorescent mitochondrial autophagy reporter protein (mito-Keima). Mitochondrial respiration was measured using high-resolution respirometry (Oroboros-O2K).

**Results:**

Addition of SA-22 altered SYPRO Orange fluorescence and improved catalytic activity *in vitro* but was abrogated by SER149 substitution, indicating that SA-22 is a positive allosteric modulator of SIRT3. Lastly, SA-22 protected cardiac cells against HR-induced changes in mitophagy and mitochondrial respiration in a SIRT3-dependent manner.

**Conclusion:**

In conclusion, SA-22 directly binds and enhances the activity of SIRT3, preserving cardiac mitochondrial homeostasis despite myocardial hypoxia-reoxygenation injury.

## 1 Introduction

For over 50 years it has been appreciated that the magnitude of myocardial tissue damage following coronary occlusion is not determined immediately at the onset of ischemia but can be altered by therapeutic interventions applied during ischemia (Maroko et al., 1971). However, despite the identification of hundreds of cardioprotective interventions, both pharmacologic and nonpharmacologic, in experimental animal models, few strategies beyond timely reperfusion have successfully translated into clinical practice (Timmis et al., 2024). Consequently, the development of ischemic heart disease (IHD) remains a significant cause of mortality and disability globally. With the total economic burden of IHD expected to continually rise, the development of efficacious treatments remains highly desirable (Ibanez et al., 2017).

While immediate reperfusion is the gold standard treatment, restoration of myocardial blood flow paradoxically induces a secondary wave of cell death known as ‘Reperfusion Injury’ (Neumann et al., 2019). Briefly, anaerobic metabolism becomes favored under ischemic conditions due to oxygen scarcity. As a result, intracellular ion homeostatic mechanisms gradually fail, culminating in mitochondrial permeability transition pore (mPTP) opening and subsequent necrotic cell death upon reperfusion and normalization of intracellular pH (Robichaux et al., 2023). Mitochondrial health is a key determinant of cellular fate following reperfusion, and previous approaches targeted towards protecting mitochondria have observed significant cardio-protection (Atici et al., 2023; Bouhidel et al., 2015; Cai et al., 2022).

Mitochondrial Quality Control (MQC) refers to the network of mechanisms which regulate and preserve mitochondrial health and homeostasis. Generally, MQC mechanisms fall into three broad categories; those which regulate mitochondrial metabolism (ETC and OXPHOS), those which facilitate the clearance of dysfunctional mitochondria and their proteins (mitophagy and proteostasis), and those which promote the generation of new healthy and functional mitochondria (mitobiogenesis). Evidence indicates that MQC processes are heavily related to cardiovascular disease (CVD) outcomes, including ischemia-reperfusion (IR) injury following myocardial infarction (Atici et al., 2023).

Silent mating-type information regulation 2 homologs (sirtuins) have gained widespread recognition as potent regulators of MQC. Catalytically dependent on the energetic co-factor nicotinamide adenine dinucleotide (NAD^+^), sirtuins influence hundreds of biochemical pathways through the removal of various acyl moieties from protein lysine residues (Tang, 2016). Research has shown that the mitochondrially-localized sirtuin family member SIRT3 has an important role in regulating mitochondrial bioenergetics through the deacetylation of metabolic enzymes (Yang et al., 2016). Furthermore, recent studies have indicated that SIRT3 is involved with cardiac MQC and exerts mitoprotective properties during myocardial ischemia-reperfusion injury (Zheng et al., 2022).

Epoxydocosapentaenoic acids (EDPs) are a group of epoxylipid metabolites that are derived from the polyunsaturated fatty acid (PUFA) docosahexaenoic acid (DHA) by Cytochrome P450 epoxygenases (CYPs) (Jamieson et al., 2017). Unfortunately, due to extensive molecular and metabolic heterogeneity amongst PUFAs, knowledge regarding the biological targets and roles of their metabolites in CVDs has remained limited (Schunck et al., 2018; Valencia et al., 2022). Subsequently, our group has worked to characterize the cardioprotective properties of both native EDPs and synthetic analogs in models of ischemia-reperfusion injury, hypoxia-reoxygenation injury, and endotoxemia (Darwesh et al., 2020; Darwesh et al., 2019; Samokhvalov et al., 2016; Samokhvalov et al., 2015). Recently, we demonstrated that the beneficial effects of the endogenous 19,20-EDP regioisomer and its structural analog, SA-22, are dependent on SIRT3 activity (Darwesh et al., 2025; Kranrod et al., 2024). This study aims to define the impact of EDP–SIRT3 signaling on mitochondrial homeostasis following *in vitro* cardiac hypoxia–reoxygenation injury.

## 2 Methods

### 2.1 Synthesis of the 19,20-EDP analog SA-22

**FIGURE 1:**
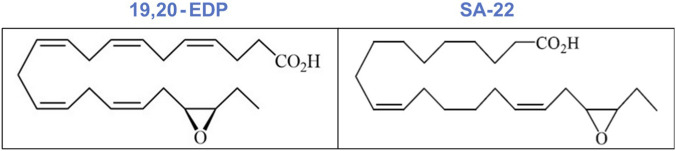
19,20-EDP as a natural CYP-Derived EpFA and its synthetic analogue SA-22.

(10Z,16Z)-18-(3-Ethyloxiran-2-yl) octadeca-10,16-dienoic acid (SA-22) was synthesized by the Falck laboratory using established synthetic methods and characterized using 1H/13C Nuclear Magnetic Resonance (NMR) and mass spectroscopy as described previously (Falck et al., 2014; Kranrod et al., 2024; Zhang et al., 2014) and 19,20‐EDP was purchased from (Cayman Chemicals) (Figure 1).

### 2.2 Cell culture and hypoxia-reoxygenation treatment protocols

H9c2 rat cardiomyoblast cells were cultivated in low glucose (1 g/L) DMEM media supplemented with 10% fetal bovine serum and 1% penicillin-streptomycin. Cell differentiation was induced by the supplementation of media with 10 nM all-trans retinoic acid for 14 days as described previously (Branco et al., 2015). Cells were seeded at a density of 1.0 × 10^6^ cells/mL and culture conditions were maintained at 37 °C, 5% CO_2_ and 95% air. Treatments included vehicle, SA-22 (1 μM), 19,20-EDP (Cayman Chemical, 10175, 1 μM), Nicotinamide (NAM, Sigma-Aldrich, N3376, 30 μM), and 3-(1H-1,2,3-triazol-4-yl) pyridine (3-TYP, Selleck, 58628, 50 μM). Stock solutions of NAM and 19,20-EDP were prepared in 100% ethanol. Stock solutions of SA-22 and 3-TYP were prepared in 100% DMSO. Final concentrations of both solvents were less than 0.1% of the treatment solutions.

All hypoxic treatments employed deoxygenated medium. Cells were placed in a computer-controlled humidified hypoxic (H) chamber (0.9% O_2_, 5% CO_2_, and 94% N_2_) for 24 h, followed by reoxygenation (R) under normoxic conditions for 6 h and the deoxygenated media was aspirated and replaced with normoxic media. Control groups were treated under normoxic conditions for 30 h. Hypoxic cells received experimental drugs upon reoxygenation with normoxic cells being treated at hour 24. The hypoxic chamber and controller were custom-designed and assembled in the instrumentation workshop at the Faculty of Pharmacy and Pharmaceutical Sciences, University of Alberta, Edmonton, AB, Canada. All *in vitro* experiments were performed with a minimum of three independent replicates. Each replicate represents a distinct sample derived from separate cell passages. Cells were passed to a maximum of 20 passages before being renewed from a frozen stock.

### 2.3 Immunoblotting

Subcellular fractions were isolated from dissociated H9c2 cell cultures as previously described (Qadhi et al., 2013). Briefly, cells were homogenized using a standard buffer (75 mM NaCl, 1 mM NaH_2_PO_4_, 8 mM NaHPO_4_, 10 mM Tris-HCl, 250 mM sucrose, and PPI). Homogenates were first centrifugated at 800 *g* for 10 min. Subsequent centrifugation of the supernatant at 10,000 *g* for 20 min produced mitochondria-enriched pellets. Mitochondrial pellets were resuspended in a homogenization buffer for analysis. Ultracentrifugation of the supernatant at 105,000 *g* for 60 min separated microsomal membranes from the cytosol.

Total protein concentration was assessed via Bradford assay, and Western blotting was carried out as described previously (Darwesh et al., 2019). Sample proteins (15–50 µg) were reconstituted in Laemmli buffer and boiled for 5 min. Samples were then loaded into Mini-PROTEAN TGX™ gels for sodium dodecyl sulfate polyacrylamide gel electrophoresis (SDS-PAGE). Proteins were resolved by SDS-PAGE (90 V, 2–3 h) within an ice-bath prior to electrophoretic transfer (25 V, overnight) to Immun-Blot™ polyvinylidene difluoride membranes. Prior to probing, membranes were blocked with tris-buffered saline tween solution (TBST; 50 mM Tris-HCl (pH = 7.6), 150 mM NaCl, 0.1% Tween-20) with 5% BSA. Following blocking, membranes were incubated overnight with primary antibodies against DRP-1 (1/1,000, Santa Cruz, sc-271583), PINK1 (1/1,000, Abcam, ab23707), Parkin (1/1,000, Cell Signaling Technology, cs2132), p62 (1/1,000, Abcam, ab5646), VDAC (1/1,000, Abcam, ab14734), and HSP60 (1/2,000, Cell Signaling Technology, cs4870). After washing, membranes were then incubated with the corresponding secondary antibodies prepared in TBST + 5% instant skim milk powder. Blots were visualized using the chemiluminescent substrate SuperSignal^TM^ West Pico PLUS and a ChemiDoc MP Imaging System. Densitometric analyses were performed using ImageJ (NIH, USA). Membranes were re-probed following incubation with stripping buffer (1.5% glycine, 0.1% SDS, 1% Tween-20, pH = 2.2) and 1 h re-blocking.

### 2.4 Lentivirus transfection and stable mito-keima expression in H9c2 cells

The pLJM1-mito-Keima transfer plasmid was constructed by subcloning the mito-Keima open reading frame (ORF) from the CoralHue® Mitochondria-targeted mKeima-Red plasmid into an empty pLJM1 vector. Transfection complexes were formed by incubating 15,000 ng pLJM1-mito-Keima plasmid, 6,000 ng pCMV-dR8.2 dvpr packaging plasmid, 3,000 ng pCMV-VSV-G envelope plasmid, and Lipofectamine 2000 Transfection Reagent in 750 μL Opti-MEM at room temperature for 30 min. Transfection complexes were incubated with wild-type HEK293T cells in T75 flasks with 10 mL DMEM supplemented with 10% FBS and 100 U/mL penicillin/streptomycin for 24 h at 37 °C and 5% CO2. Cell culture media was replaced the following day. Lentivirus-containing DMEM was collected after another 24 h. Cells were cleared by centrifugation at 500 *g* for 5 min followed by filtration through a 0.45 μm PES syringe filter. H9c2 cells were seeded in 6-well plastic culture plates and grown to 60% confluency before the culture media was replaced with 2 mL of lentivirus-containing DMEM. Cells were spinfected by centrifuging 6-well plates at 1,000 × g for 45 min at 37 °C. Media was replaced with fresh DMEM after 24 h. The following day, culture media was supplemented with 2 μg/mL puromycin. Once cells reached 80%–90% confluency they were seeded into larger dishes for further cultivation and frozen in liquid nitrogen for long-term cryostorage (Valencia et al., 2024). Stable mito-Keima expression was validated using fluorescence microscopy. pLJM1-EGFP was a gift from David Sabatini (Addgene plasmid #19319; http://n2t.net/addgene:19319; RRID:Addgene_19319). pCMV-dR8.2 dvpr was a gift from Bob Weinberg (Addgene plasmid#8455; http://n2t.net/addgene:8455; RRID:Addgene_8455). pCMV-VSV-G was a gift from Bob Weinberg (Addgene plasmid#8454; http://n2t.net/addgene:8454; RRID:Addgene_8454).

### 2.5 Microscopy

#### 2.5.1 Live-cell fluorescence microscopy

For fluorescent microscopy experiments, H9c2 cells were plated on 6-well glass bottom plates and subjected to HR injury as described above. Cells were incubated with 1 µM Hoechst 33342 dye to identify nuclear DNA. In all experiments, cells were imaged using a Zeiss Axio Observer Z1 inverted epifluorescence microscope fitted with a micro incubator (37 °C, 5% CO2), using a 40X/1.3NA oil DIC objective lens. Images were captured using the Zeiss Zen© 2.3 software).

### 2.6 Quantification of mito-keima fluorescence

Fluorescence images were generated according to the following parameters: Neutral pH mitochondria fluorescence was excited at 470 nm (25% intensity, 750 ms exposure) and captured at 604 nm. Acidic pH mitochondrial fluorescence was excited at 590 nm (80% intensity, 800 ms exposure) and captured at 604 nm. Hoechst 33342 (DNA) fluorescence was excited at 365 nm (10%, 100 ms exposure) and captured at 455 nm. Acidic object counts were normalized to nuclei counts manually and experimenters were blinded to treatments.

### 2.7 Assessment of SIRT3 deacetylation activity

We assessed the effect of 19,20-EDP and SA-22 on hSIRT3 activity *in vitro* using a commercial fluorogenic HDAC assay kit (BPS Bioscience, 50088). This assay involves incubation of either recombinant hSIRT3 or biological samples with a specific fluorogenic HDAC substrate, bovine serum albumin, NAD^+^, and a proprietary assay buffer. Upon initial deacetylation by SIRT3 and subsequent exposure to the assay developer solution, the HDAC substrate produces a fluorescent product that can be measured using a fluorometric plate reader at 380/460 nm excitation/emission wavelengths. SIRT3 activity was expressed as a fold of the aerobic vehicle in relative luminescence units (RLU). 2 μg of hSIRT3 was added to each reaction.

### 2.8 Human SIRT3 plasmid site-directed mutagenesis, expression in *E. coli*, and protein purification

SIRT3L-12, a bacterial protein expression plasmid containing a 6xHis-tagged human SIRT3 (hSIRT3) open reading frame (ORF), was a gift from John Denu (Addgene plasmid # 13736; http://n2t.net/addgene:13736; RRID: Addgene_13736). Mutagenic primers for the alanine substitution S149A were designed using the NEBasechanger web tool (https://nebasechanger.neb.com/) (20). The Q5® Site-Directed Mutagenesis Kit (New England BioLabs, Ipswich, MA, USA) was used to create S149A SIRT3L-12. S149A SIRT3L-12 was transformed into Subcloning Efficiency DH5α Competent Cells (Invitrogen, Thermo Fisher Scientific, Inc., Waltham, MA, USA). Select colonies were cultured using the QIAprep Spin Miniprep Kit (QIAGEN, Hilden, Germany) according to kit protocol along with 100 μg/mL carbenicillin. Presence of intact ORFs with the S149A substitution was confirmed via Sanger sequencing at the Molecular Biology Service Unit in the Department of Biological Sciences at the University of Alberta.

Both wildtype and S149A SIRT3L-12 were transformed into BL21 (DE3) Competent *E. coli* cells (New England BioLabs, Ipswich, MA, USA). Individual colonies were grown overnight in 10 mL Luria Bertani broth (LB)-containing antibiotics at 37 °C and 220 RPM. The next day, 1 mL of cell culture was added to 100 mL of fresh LB and induced with 1 mM IPTG when OD600 had reached 0.6 (approx. 1.5 h). Cells were kept at 37 °C and 220 RPM for another 4–5 h post-induction. Cell cultures were collected via centrifugation at 6,000 × *g* and 4 °C for 20 min. Pellets were resuspended in 10 mL of aqueous 10 mM Tris-HCl (pH = 8.0) buffer. Cells were re-pelleted and resuspended in 4 mL B-PER® Bacterial Protein Extraction Reagent (Thermo Fisher Scientific, Inc., Waltham, MA, USA) and incubated at room temperature for 15 min. Lysates were centrifuged at 10,000 × *g* for 15 min at 4 °C. Supernatant was collected and diluted with 10 mL of 10 mM Tris-HCl (pH = 8.0) before being incubated with 0.5 mL of 50% Ni2+-NTA agarose slurry at 4 °C with rotary shaking for 1 h. A centrifugation at 5,000 × *g* and 4 °C for 5 min removed unbound proteins. Two subsequent washes with 10 mL of 10 mM Tris-HCl (pH = 7.5) containing 10 mM imidazole removed non-specifically bound proteins. Bound protein was sequentially eluted with 1 mL fractions of 50 mM and 250 mM imidazole in Tris-HCl (pH = 8.0). Elution fractions were analyzed with 12% SDS-PAGE with fractions possessing recombinant hSIRT3 were pooled and concentrated using Amicon® Ultra-4 10K Centrifugal Filter Units (MilliporeSigma, Burlington, MA, USA).

### 2.9 Thermal shift assay

hSIRT3-SA-22 binding was measured via thermal shift assay with the SYPROⓇ Orange protein dye (Nettleship et al., 2008). 10 μM of wild-type or S149A mutant protein, 20X SYPRO Orange dye, and 100 µM SA-22 or equivalent amount of vehicle (DMSO) were diluted in 50 mM Tris-HCl (pH = 8.0) and 50 mM NaCl assay buffer to a final reaction volume of 20 µL (Darwesh et al., 2025). Thermal shift assays (TSA) were performed in a BioRad CFX Connect Real-Time PCR Detection System using the FAM dye reporter filter set and melt-curve analysis (25 °C for 2 min followed by melt-curve from 25 °C to 95 °C with 0.5 °C/10 s steps). First derivatives of the raw fluorescence values were plotted, and the temperature with the maximum rate of change in fluorescence was taken as the melting temperature (T-m). T-m shifts induced by drug treatments (delta T-m) were obtained by subtracting the T-m of the respective vehicle control group.

### 2.10 High-resolution mitochondrial respirometry

Mitochondrial respiratory function was assessed in H9c2 cells using an OroborosO2k high-resolution respirometer (OROBOROS Instruments, Innsbruck, Austria) according to the substrate-uncoupler-inhibitor titration (SUIT) protocol 008 (Lemieux et al., 2017). Following experimental treatment protocols, cells were washed twice with PBS then dissociated with TrypLE™ Express enzyme. After neutralizing TrypLE™ with fresh DMEM and collecting cell suspensions, cell density was calculated using the trypan-blue exclusion assay. Remaining cells were centrifuged at 500 × *g* for 4 min. Pellets were resuspended in mitochondrial respiration medium (MiR05, pH = 7.1, 0.5 mM EGTA, 3 mM MgCl2, 60 mM potassium lactobionic acid, 20 mM taurine, 10 mM KH2PO4, 20 mM HEPES, 110 mM D-sucrose, 1 g/L fatty acid-free BSA, dissolved in double-distilled water). 2.0 mL of cell suspension was then added to closed chambers at 37 °C and 750 RPM stirrer speed. Cells were given 20 min to equilibrate within the chambers before the basal respiratory state was recorded. Selective permeabilization of cytosolic membranes was achieved by incubating cells with 4 µM digitonin for 20 min prior to commencement of the SUIT protocol. Oxygen consumption rates (OCRs) at each respiratory state were recorded in the following order: (i) LEAK respiration (oxygen consumption not coupled to ATP production) stimulated by 5 mM pyruvate and 2 mM malate [PML]; (ii) OXPHOS stimulated by 2.5 mM adenosine diphosphate (ADP) [PMP]; (iii) Mitochondrial outer membrane integrity was tested by addition of 10 µM cytochrome c [PMcP]; (iv) Complex I respiration was saturated with 10 mM glutamate [PMGP]; (v) Maximal complex I+II OXPHOS respiration following 10 mM succinate addition [PMGSP]; (vi) Maximal non-coupled respiration (ET) following several step-wise 0.1 µM FCCP titrations [PMGSE]; (vii) Non-coupled complex II respiration with 0.5 µM rotenone [SE]; (viii) Residual oxygen consumption (ROX) after 2.5 µM antimycin A titration (Lemieux et al., 2017). Reported OCRs were calculated by normalizing all O2 flux values to ROX respiration and cell count ([pmol O2]/[sec*million cells]).

### 2.11 Statistical analysis

Statistical analyses were conducted using GraphPad Prism software (Version 10.2.0 (392)). Values are presented as mean ± standard error of mean (SEM). Statistical significance was determined by one-way ANOVA with a Bonferroni *post hoc* test when comparing two or more groups to a control mean; p < 0.05 was considered statistically significant. Two-way ANOVA with a bonferroni *post hoc* test for multiple comparisons; P < 0.05. The Shapiro-Wilk Normality test has been carried out to confirm that the data is parametric. Individual points on data plots/graphs represent separate biological replicates.

## 3 Results

### 3.1 SA-22 directly binds and potentiates SIRT3 catalytic activity *in vitro*

**FIGURE 2:**
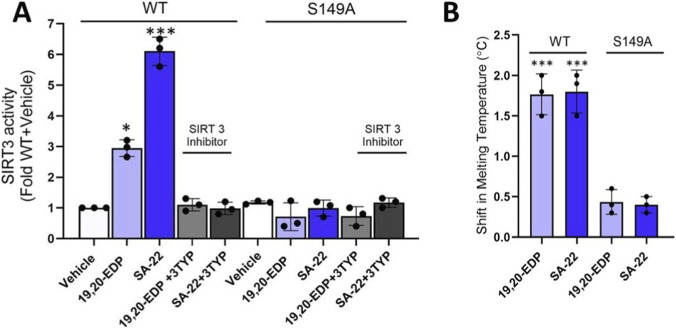
SA-22 Directly Binds SIRT3 and improves catalytic activity *in vitro*. **(A)** Fluorogenic SIRT3 assay reaction conducted in the presence or absence of 19,20-EDP, SA-22, and 3-TYP. Data is presented in relative luminescence units (RLU) as a fold change compared to vehicle treatment. Values represent mean ± SEM, *p < 0.05 vs. aerobic vehicle (n = 3 per group). Treatments: vehicle (0.1% v/v DMSO), 19,20-EDP (1 or 100 µM), SA-22 (1 or 100 µM), and 3-TYP (50 µM). SA-22; (10Z,16Z)-18-(3-Ethyloxiran-2-yl)-octadeca-10,16-dienoic acid. 3-TYP; 3-(1H-1,2,3-triazol-4-yl) pyridine. 19,20-EDP; 19,20-Epoxydocosapentaenoic Acid. SIRT3; silent mating type information regulation 2 homolog. **(B)** SYPRO Orange thermal shift assay where recombinant SIRT3 (wildtype or S129A) was treated *in vitro* with either 19,20-EDP or SA-22. Data is presented as the absolute change in peak melting temperature relative to vehicle. Values represent mean ± SEM, *p < 0.05 vs. aerobic control (n = 3 per group).

To determine whether SA-22 could bind and alter the catalytic activity of SIRT3 *in vitro* we employed both a fluorogenic SIRT3 activity assay and a thermal shift assay. In addition to ‘wildtype’ hSIRT3, we produced mutant hSIRT3 possessing the S149A substitution, previously demonstrated by our group to be catalytically active but insensitive to 19,20-EDP (Darwesh et al., 2025). After our previous report that SA-22 was similarly dependent on mitochondrial SIRT3 activity for its salutary effects, we aimed to determine whether the salutary effects of SA-22 were also reliant on interactions with the S149 residue (Kranrod et al., 2024). Interestingly, while both 19,20-EDP and SA-22 significantly increased the catalytic activity of hSIRT3, SA-22 significantly outperformed 19,20-EDP in this respect (Figure 2A). Co-treatment with 3-TYP or substitution of S149 with alanine completely abrogated the positive effect of both 19,20-EDP and SA-22 on hSIRT3 activity (Figure 2A). Encouragingly, 19,20-EDP and SA-22 also significantly shifted the peak melting temperature of hSIRT3 according to SYPRO® Orange assay. Crucially, this effect too was inhibited when S149A hSIRT3 was substituted for wildtype enzyme (Figure 2B).

### 3.2 SA-22 protects cellular respiratory capacity ETC efficiency

**FIGURE 3:**
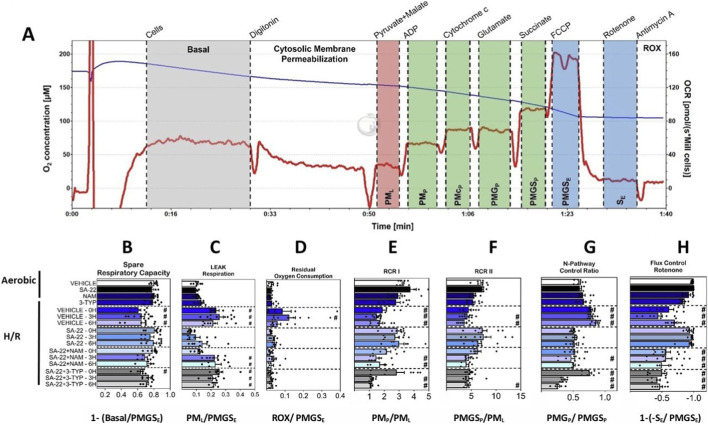
SA-22 preserves mitochondrial respiratory function dependent on SIRT3 *in vitro* following HR challenge. High-resolution respirometry-based analysis (SUIT-008) of H9c2 cells *in vitro* was performed following HR in the presence or absence of SA-22, NAM, and 3-TYP. **(A)** Representative high-resolution respirometry experiment. Oxygen consumption rates (OCRs) are measured at different states induced by various substrates, uncouplers, and inhibitor titrations. Resulting oxygen consumption ratios include spare respiratory capacity (SRC) Blue trace indicates oxygen concentration (µM), while the red trace indicates oxygen consumption rate (OCR; pmol·s^-1^·10^6^ cells^-1^). **(B)**, LEAK respiration **(C)**, residual oxygen consumption **(D)**, respiratory control ratio (RCR) for complex I and II **(E,F)**, N-Pathway control ratio **(G)**, and the uncoupled flux control efficiency for complex I **(H)**. Treatments: vehicle (0.1% v/v DMSO), SA-22 (1 µM), NAM (30 µM), and 3-TYP (50 µM). Values represent mean ± SEM, #p < 0.05 vs. aerobic control (n = 5–18 per group). SA-22; (10Z,16Z)-18-(3-Ethyloxiran-2-yl)-octadeca-10,16-dienoic acid. NAM; Nicotinamide. 3-TYP; 3-(1H-1,2,3-triazol-4-yl) pyridine. HR; hypoxia-reoxygenation. ROX; Residual Oxygen Consumption. SRC; Spare Respiratory Capacity. RCR; Respiratory Control Ratio. SUIT; Substrate-Uncoupler-Inhibitor Titration. P; Pyruvate. M; Malate. G; Glutamate. S; Succinate. L; LEAK respiration. E; maximum uncoupled respiration. P; ADP-stimulated respiration.

To investigate SA-22-mediated mitochondrial protection we employed high-resolution respirometry (Oroboros-O2k) to assess mitochondrial function (Figure 3; Table 1). A trace from a representative experiment (SUIT-008) is shown for illustrative purposes (Figure 3A). Raw OCRs recorded at the intervals highlighted in Figure 3A are provided (Table 1). Various measures of mitochondrial function were then derived from cellular OCRs (Figure 3B). HR injury reduced oxygen consumption compared to aerobic vehicle control at all recorded intervals until the addition of rotenone following uncoupling of the ETC with FCCP treatment (Table 1). In contrast, cells treated with SA-22 consumed oxygen at a comparable rate to aerobic control even when subjected to hypoxic insults. Furthermore, co-treatment with either NAM or 3-TYP largely abrogated these benefits (Table 1). Analysis of the OCR data provided further insight; HR injury reduced maximal mitochondrial respiratory capacity (presented as spare respiratory capacity (SRC)), suggesting ETC impairment, which was protected by addition of SA-22 (Figures 3B–H). Inhibition of SIRT3 with co-treatment of either NAM or 3-TYP abrogated SA-22’s protective effects. When normalized to total ETC capacity, HR vehicle-treated cells displayed elevated levels of LEAK respiration, implying that a greater proportion of their respiratory capacity is dedicated to compensating for proton and electron leakage, suggesting disruption of the ETC complexes and of the mitochondrial matrix (Figure 3C), which was ameliorated by SA-22. Residual oxygen consumption (ROX) is a total measure of the oxidative side reactions occurring in cells not coupled to ATP production (Keilin, 1966). While this may be an effect of an overall decreased respiratory capacity, HR significantly increased ROX with SA-22 again exhibiting protective properties (Figure 3D). Dependent on SIRT3 activity, SA-22 preserved both complex I and II respiration (presented as RCR I and II respectively) stimulated by ADP and succinate respectively when compared to HR vehicle (Figures 3E,F). Interestingly, pharmacological SIRT inhibition caused a time-dependent decrease of complex I respiration which corresponds with a time-dependent increase in LEAK respiration (Figure 3E). The NS-Pathway Control Ratio is a measure of the relative contribution of both complex I (NADH-linked substrates; N-Pathway) and complex II (Succinate; S-Pathway) to convergent flux through the Q-junction (Lemieux et al., 2017). Deviation from a recorded physiological normal ratio or baseline may potentially indicate a deficiency in the activity of a specific complex. Paradoxically, while HR vehicle groups displayed significantly higher complex I-mediated control over NS-pathway respiration prior to uncoupling with FCCP, rotenone inhibited oxygen consumption in hypoxic groups to a much lesser degree when compared to aerobic control and SA-22-treated hypoxic cells after uncoupling (Figures 3G,H).

**TABLE 1:** Raw oxygen consumption rates in H9c2 cells treated with SA-22, NAM and TYP.

State	Aerobic				HR- 6Hours			
Groups	VEH	SA-22	NAM	3-TYP	VEH	SA-22	SA-22+NAM	SA-22+3-TYP
Basal	49.2 ± 1.5	56.8 ± 2.6	51.3 ± 1.4	36.8 ± 4.3*	33.7 ± 3.7*	45.8 ± 3.1	26.3 ± 1.4*	28.0 ± 2.3*
PM_L_ (N_L_)	27.3 ± 4.1	25.5 ± 1.6	29.4 ± 1.2	22.7 ± 1.3	20.6 ± 1.2	31.0 ± 2.9	21.2 ± 1.4	22.4 ± 2.9
PM_p_ (N_P_)	90.0 ± 16.2	96.1 ± 13	84.5 ± 8.5	64.3 ± 8.6	35.1 ± 5.0*	83.6 ± 5.8	35.6 ± 5.9*	26.0 ± 3.5*
PMc_P_ (N_P_)	92.9 ± 11.7	109.2 ± 6.9	115.8 ± 8.3	94.5 ± 8.5	39.2 ± 6.2	97.1 ± 15.3	38.8 ± 8.4	24.6 ± 3.5*
PMG_p_ (N_P_)	105.9 ± 10.1	110.9 ± 7.3	102.7 ± 7.2	82.1 ± 11.3	63.4 ± 5.9*	98.8 ± 10.7	43.6 ± 5.1*	25.4 ± 3.5*
PMGS_P_ (NS_P_)	184.4 ± 24.0	183.5 ± 8.0	158.5 ± 11.9	121.7 ± 15.5	73.9 ± 7.3*	188.4 ± 13.3	84.5 ± 8.8*	94.1 ± 15.2*
PMGS_E_ (NS_E_)	236.7 ± 26.7	261.3 ± 23.2	277.6 ± 19.7	182.2 ± 23.5	106.8 ± 13.6*	270.4 ± 42.5	100.1 ± 13.2*	102.0 ± 13.5*
PMGS(Rot)_E_ (S_E_)	5.0 ± 1.0	1.5 ± 0.4	22.2 ± 7.9	30.3 ± 3.9	32.7 ± 5.4	25.6 ± 17.5	50.3 ± 15.1	55.3 ± 10.1
ROX	7.7 ± 1.5	6.0 ± 1.3	5.4 ± 0.9	5.4 ± 1.3	6.1 ± 1.7	12.3 ± 2.6	2.2 ± 0.6	3.4 ± 0.9

High-resolution respirometric assessment of H9c2 cells subjected to HR insult with or without SA-22, NAM, and 3-TYP. Following permeabilization with digitonin (4 µM), cells were analyzed with the SUIT-008 protocol. OCRs from each respiratory state were obtained by normalizing oxygen flux following each substrate titration to ROX; Basal, Pyruvate (5 µM) and Malate (2 µM) (PML), ADP (10 µM) (PMP), Cytochrome c (10 µM) (PMcP), Glutamate (10 µM) (PMGP), Succinate (10 µM) (PMGSP), FCCP (0.3 µM) (PMGSE), Rotenone (0.5 µM) (PMGS (Rot)E), and Antimycin A (2.5 µM) (ROX). Treatments; vehicle (0.1% v/v DMSO), SA-22 (1 µM), NAM (30 μM), and 3-TYP (50 μM). Values represent mean ± SEM (n = 4–18 per group). SA-22; (10Z,16Z)-18-(3-Ethyloxiran-2-yl)-octadeca-10,16-dienoic acid. NAM; Nicotinamide. 3-TYP; 3-(1H-1,2,3-triazol-4-yl) pyridine. FCCP; Carbonyl cyanide p-trifluoromethoxyphenylhydrazone. HR; hypoxia-reoxygenation. ROX; Residual Oxygen Consumption. P; Pyruvate. P; ADP-stimulated respiration. M; Malate. G; Glutamate. S; Succinate. L; LEAK. E; Maximum uncoupled respiration. N; N-Pathway (i.e., Complex I). OCR; oxygen consumption rate. SUIT; Substrate-uncoupler-inhibitor titration.

* p < 0.05 vs. aerobic control.

### 3.3 SA-22 and mitophagy post-HR injury

Following our observation that SA-22 preserved electron transport chain (ETC) activity after HR injury, we next examined whether SA-22 could influence cardiac mitochondrial integrity. Specifically, whether SA-22 bioactivity involves modulation of mitophagy. Mitophagy is an MQC mechanism wherein damaged mitochondria are selectively marked (such as via ubiquitination) for autophagic degradation (Lemasters, 2005). As preventing the accumulation of damaged mitochondria is crucial for maintaining cellular health, mitophagy is a vital process for cellular homeostasis. Furthermore, contemporary studies have recognized the important role of mitophagy during ischemic myocardial injury (Gottlieb et al., 2011). Given that mitophagy is a dynamic and time-dependent process, we employed H9c2 cells stably expressing mito-Keima, a mitochondrially targeted, lysosome-resistant, pH-sensitive fluorescent reporter that enables quantification of mitophagic flux (Katayama et al., 2011).

**FIGURE 4:**
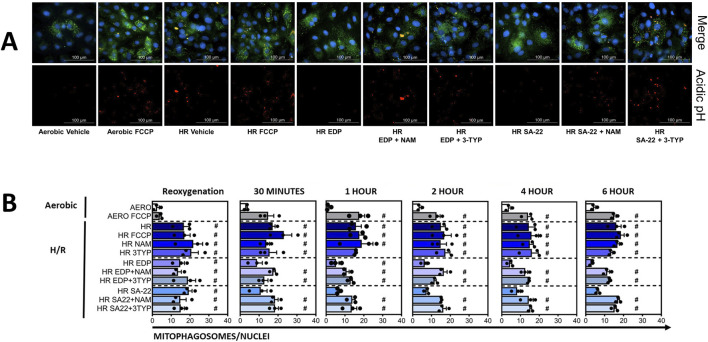
Preservation of mitophagy post-HR by SA-22 is SIRT-dependent. Mitophagosome levels were assessed using mito-Keima, a mitochondrially-targeted, lysosome-resistant, fluorescent protein with pH-dependent excitation spectra. FCCP is used as a positive control for mitophagy, and VDAC is used as a loading protein, 3-TYP as SIRT3 inhibitor. **(A)** Representative fluorescence images from H9c2 cells stably expressing mito-Keima were treated with either vehicle, FCCP, 19,20-EDP, SA-22, or co-treatment of either SA-22 with either NAM, or 3-TYP. Photos were taken 6-h post-reoxygenation. White bar = 100 μm. **(B)** Quantification of mitophagosome levels (normalized to nuclei count) at several timepoints following reoxygenation including at reoxygenation, 30 min, 1, −2, −4, and 6 h post-reoxygenation. Aerobic control images were taken at equivalent times in the absence of previous hypoxic challenge. Treatments; vehicle (0.1% v/v DMSO), FCCP (10 μM), 19, 20-EDP (1 μM), NAM (30 μM), 3-TYP (50 μM), or SA-22 (1 μM). SA-22; (10Z, 16Z)-18-(3-Ethyloxiran-2-yl)-octadeca-10, 16-dienoic acid. EDP; Epoxydocosapentaenoic acid. 3-TYP: 3-(1H-1, 2, 3-triazol-4-yl) pyridine. NAM; Nicotinamide. HR; hypoxia-reoxygenation. FCCP; Carbonyl cyanide p-trifluoromethoxyphenylhydrazone, #p < 0.05 vs. aerobic control.

Immediately following reoxygenation, all hypoxic groups possessed an elevated number of ‘mitophagosomes’ (Figure 4). Interestingly, by 1-h post-reoxygenation, both the 19,20-EDP and SA-22 treated groups had significantly fewer mitophagosomes compared to the HR vehicle group (Figure 4). At the end of the 6-h reoxygenation period, mitophagosome levels in the EDP and analog-treated groups had returned to near aerobic levels (Figure 4). In contrast, the groups that received co-treatment of 19,20-EDP or SA-22 with a sirtuin inhibitor (NAM or 3-TYP), showed no improvement in the number of mitophagosomes, demonstrating that this effect was also SIRT3-dependent. Normoxic cells treated with 19,20-EDP, SA-22, NAM, or 3-TYP did not demonstrate any changes in mitophagy (Figure 4). Representative images from the 6-h post-reoxygenation timepoint were provided to illustrate the mitoprotective effect of SA-22 treatment against HR injury. In the merged images, healthy and damaged mitochondria are coloured green and red respectively, with DNA being coloured blue. In addition, the fluorescence images corresponding solely to mitochondria at acidic pHs (red) are presented separately (Figure 4). Treatment with either SA-22 or 19,20-EDP prevented the accumulation of acidic mitochondria observed in cells subjected to HR injury. Pharmacological inhibition of SIRT3 abrogated these benefits (Figure 4).

**FIGURE 5:**
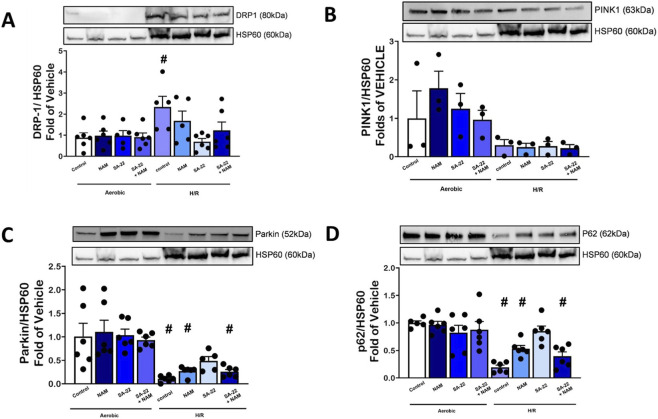
SA-22 treatment significantly ameliorated the deficient localization of Parkin and p62 to mitochondria in a SIRT-dependent manner. Mitochondrial localization proteins are assessed by using Western blot technique. NAM is used as a sirtuin inhibitor, HSP60 is used as a loading protein. Data was analyzed using one-way ANOVA followed by Bonferroni as a *post hoc* test and expressed as mean ± SEM, #p < 0.05, n = 4–6. **(A)** Quantitative expression of DRP-1 relative to HSP60 protein. **(B)** Quantitative expression PINK1 relative to HSP60 protein. **(C)** Quantitative expression PARKIN relative to HSP60 protein. **(D)** Quantitative expression P62 relative to HSP60 protein.

Following HR injury, cells were assessed for changes to proteins involved in mitophagy. There was a marked increase of mitochondrially-localized DRP-1, suggesting that damaged mitochondria were being targeted for removal after 6 h of reoxygenation (Figure 5A). Intriguingly, other markers that represent the progression and execution of mitophagy such as Parkin and p62 were significantly decreased (Figures 5B–D). Importantly, SA-22 treatment significantly ameliorated the deficient localization of Parkin and p62 to mitochondria in a SIRT-dependent manner (Figures 5C,D). SA-22 did not appear to influence mitochondrial-localization of PINK1 (Figure 5B).

**FIGURE 6:**
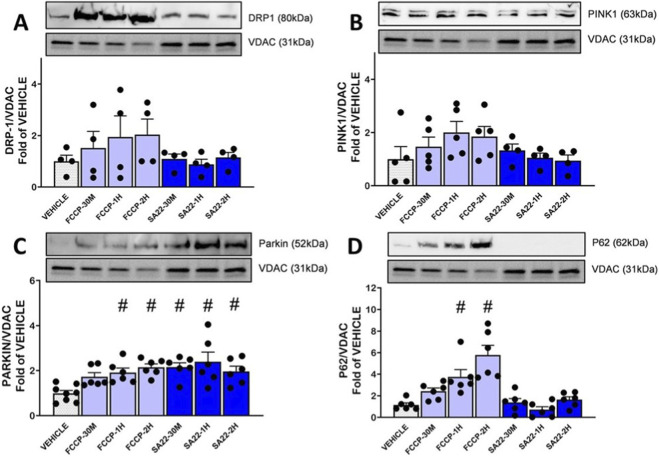
SA-22 did not trigger a mitophagic response in absence of hypoxic injury except for Parkin. Mitochondrial localization proteins are assessed by using Western blot technique. FCCP is used as a positive control for mitophagy, and VDAC is used as a loading protein. Data was analyzed using one-way ANOVA followed by Bonferroni as a *post hoc* test and expressed as mean ± SEM, #p < 0.05, n = 4–6. **(A)** Quantitative expression of DRP-1 relative to VDAC protein. **(B)** Quantitative expression PINK1 relative to VDAC protein. **(C)** Quantitative expression PARKIN relative to VDAC protein. **(D)** Quantitative expression P62 relative to VDAC protein.

To confirm whether SA-22 triggers a mitophagic response irrespective of cellular injury, we treated non-challenged H9c2 cells with either SA-22 or FCCP (positive control for mitophagy) and harvested them at several times (30-min, 1-h, and 2-h) (Berezhnov et al., 2016). Immunoblot analysis of heavy membrane-enriched fractions suggests that SA-22 does not significantly trigger the mitochondrial-localization of several markers associated with mitophagy such as DRP-1, PINK1, and p62 (Figures 6A,B,D). Interestingly, SA-22 treatment appears to cause significant mitochondrial localization of Parkin, independent of any of the other assessed markers (Figure 6C).

## 4 Discussion

The precise molecular mechanism responsible for the cardioprotective effects of CYP-derived n-3 PUFA epoxylipids remains undefined despite numerous studies (Schunck et al., 2018). This work presents evidence suggesting that both 19,20-EDP and its synthetic analog SA-22 exert cardioprotective action by protecting mitochondrial health against hypoxic insult *via* activation of SIRT3. SIRT3, a member of the sirtuin family of NAD^+^-dependent deacetylases, primarily localizes to the mitochondria and regulates numerous proteins involved in mitochondria function. In particular, the deacetylation activity of SIRT3 has been demonstrated to improve the catalytic activity of mitochondrial respiratory enzymes and many others involved in energy metabolism, specifically the TCA cycle and fatty acid metabolism (Baeza et al., 2016; Cimen et al., 2010; Yang et al., 2016). Furthermore, SIRT3 deficiency has been readily shown to be deleterious with respect to cardiovascular outcomes in various experimental models (Koentges et al., 2015; Porter et al., 2014; Sundaresan et al., 2009; Yu et al., 2017). Notably, decreased expression of SIRT3 is an observed characteristic of human heart failure (Zhang et al., 2018). Impaired SIRT3 activity is thought to result in lysine hyperacetylation of mitochondrial proteins, thereby impeding cellular metabolism and respiration, contributing to energetic stress and heart failure (Baeza et al., 2016). Therefore, SIRT3 has emerged as a promising therapeutic target for various CVDs.

The current data demonstrated both 19,20-EDP and SA-22 bind hSIRT3 and positively regulate its catalytic activity. In alignment with a previous study, we observed that Serine 149 (S149) is a crucial residue for EDP-mediated activation of hSIRT3, as substitution with alanine completely blocked the positive effect of SA-22 on hSIRT3 catalysis (Darwesh et al., 2025). Interestingly, while we presented the thermal shift data as an absolute temperature change, SA-22, in contrast to 19,20-EDP, causes the peak melting temperature of hSIRT3 to change negatively. While the typical conceptualization of thermal shift assays predicts that ligands will stabilize their interaction partners and subsequently cause a positive shift in peak melting temperature, it has been demonstrated that certain ligands can cause a negative thermal shift when binding proteins in a partially unfolded state (Cimen et al., 2010). Further study is necessary for speculation on the significance of this observation.

Myocardial mitochondria continuously consume large quantities of oxygen to produce the necessary quantities of ATP for sustained myocardial contractile function. As such, human life relies upon optimal function of the mitochondrial ETC and its constituent subunits. Relative levels of SIRT3 activity have been shown to directly correlate with the respiratory capacity and ATP production of cardiac tissue (Ahn et al., 2008; Schlicker et al., 2008). Additionally, SIRT3 is known to directly associate with mitochondrial complex V, the enzyme ultimately responsible for generating ATP, and improve its activity via deacetylation. Conversely, inhibited SIRT3 activity has been shown to result in downregulation of complex V activity and ATP production (Hirschey et al., 2010). Previously our group observed that 19,20-EDP, dependent on activation of SIRT3, upregulated mitochondrial respiration and oxygen consumption in both normal and infarct heart tissue (Darwesh et al., 2025). Supporting our previous findings, the high-resolution respirometry data presented here indicates that SA-22, a synthetic analog of 19,20-EDP, preserves complex I and II activity post-reoxygenation in cardiac cells. This is accompanied by a significant reduction in LEAK respiration, indicating that SA-22 treatment protects mitochondrial membranes against disruption and enables ETC enzymes to properly organize such that electron leakage is minimized, even in the context of hypoxia-reoxygenation. Efficient complex I respiration and limiting of electron/proton leakage is crucial for minimizing mitochondrial ROS production, which is a key contributor to oxidative damage (Parodi-Rullan et al., 2017; Power et al., 2016). The broader literature indicates that the individual complexes of the respiratory chain frequently assemble into super-complexes, often dubbed ‘respirasomes’, which are marked by significant increases in respiratory efficiency (Bianchi et al., 2004; Guo et al., 2016). Conversely, when respirasome assembly is inhibited or disrupted, marked increases in ROS production can be observed (Rosca et al., 2008). Interestingly, increases in ETC complex acetylation induced by SIRT3 KO in cardiac cells have been correlated with respirasome disassembly (Parodi-Rullán et al., 2019). However, the precise mechanisms by which SIRT3 activation influences mitochondrial biology during HR injury remain to be determined.

Despite the strengths of this study, several limitations should be acknowledged. First, our *in vitro* experiments utilized H9c2 cells, which, although widely used, do not fully recapitulate the structural, metabolic, and functional characteristics of adult ventricular cardiomyocytes. While no cytotoxic effects of 19,20-EDP or SA-22 were observed under the conditions used here or in our prior *ex vivo* work, we have previously reported ceramide-dependent toxicity of 19,20-EDP in H9c2 cells cultured under high-glucose conditions (Endo et al., 2018). Subsequent studies have therefore employed low-glucose media to mitigate this effect. Given that a highly glycolytic phenotype is not representative of terminally differentiated cardiomyocytes, we anticipate that such toxicity is less likely under physiological conditions; however, caution remains warranted when extrapolating these findings to the intact heart. Second, although experiments were conducted across independent replicates, certain datasets, particularly immunoblot analyses (e.g., Figures 4A,B, 5B), exhibited variability and relatively small sample sizes, which may influence interpretation. Third, this study did not assess potential sex-dependent effects of PUFA-derived epoxylipids, an important consideration given known sex differences in cardiovascular biology. Finally, the use of a hypoxia–reoxygenation model differs from true ischemia–reperfusion *in vivo*, where reduced or absent blood flow introduces additional complexity, including altered substrate delivery, metabolite accumulation, and mechanical stress. These differences in the local environment may influence mitochondrial responses and the extent of injury. Collectively, these limitations underscore the need for future studies in more physiologically relevant systems, including primary cardiomyocytes and *in vivo* models, to further define the therapeutic potential of SA-22.

As mitophagy is an integral part of a cell’s ability to maintain mitochondrial quality, mitophagosome quantities were assessed at several timepoints following re-oxygenation. Treatment with SA-22 or 19,20-EDP produced a gradual, largely linear reduction in the number of acidic mitochondria beginning at the onset of re-oxygenation, an effect that was dependent on SIRT3 activity. These findings contrast with prior reports demonstrating robust SIRT3-mediated stimulation of mitophagy following HR injury in cardiomyocytes (Hu et al., 2022; Yang et al., 2022). Consistent with this observation, immunoblot analyses of mitophagy-related protein localization performed at 6 h post-reoxygenation revealed that SA-22, in a SIRT3-dependent manner, mitigated HR-induced alterations in mitophagy signaling. Notably, SA-22 failed to induce appreciable changes in mitochondrial acidification or mitophagy marker localization in the absence of HR stress, suggesting that its protective effects are unlikely to arise from direct enhancement of mitophagic flux. Instead, these data support a context-dependent role for SA-22 in preserving mitochondrial integrity following hypoxia–reoxygenation injury.

SIRT3 deacetylase activity promotes the maintenance of a healthier, more functional mitochondrial population that is less likely to be targeted for mitophagic removal following re-oxygenation. Consistent with this view, we have previously demonstrated that perfusion of isolated hearts with 19,20-EDP or SA-22 confers multiple mitochondrial benefits, including upregulation of antioxidant systems such as thioredoxin and enhanced deacetylation of manganese superoxide dismutase (MnSOD), thereby facilitating the scavenging and clearance of damaging reactive oxygen species that drive mitochondrial injury (Darwesh et al., 2020; Darwesh et al., 2025; Kranrod et al., 2024; Qiu et al., 2010). In addition, we have also demonstrated that perfusion with 19,20-EDP and SA-22 prevents the proteolytic cleavage of the vital mitochondrial structural protein OPA-1, which has been shown to promote mitophagy (Darwesh et al., 2020; Han et al., 2025; Kranrod et al., 2024).

In this study, we examined the effects of SA-22, a novel synthetic structurally mimetic analog of the epoxylipid 19,20-EDP, using an experimental model of cellular cardiac hypoxia-reoxygenation. We hypothesized that SA-22 protects cardiomyocytes against HR injury by preserving mitochondrial homeostasis via potentiation of SIRT3 activity. This hypothesis is strongly supported by our findings, as many of the protective effects of SA-22 were lost upon co-treatment with SIRT3 inhibitors. Although further investigation into the vital aspects of EDP-mediated SIRT3 bioactivity in the context of myocardial ischemia is required, this study reinforces our previous conclusions about the therapeutic potential of synthetic EpFA analogs as anti-ischemic pharmacological agents.

## Data Availability

The raw data supporting the conclusions of this article will be made available by the authors, without undue reservation.
