# Supplementary figures and images for: Establishing a direct interaction between the 19,20-EDP analog SA-22 and SIRT3: impact on cardiac mitochondrial homeostasis
Source: Front Pharmacol. 2026 Jun 2;17:1805965. doi: 10.3389/fphar.2026.1805965 (PMC13268912; doi:10.3389/fphar.2026.1805965)

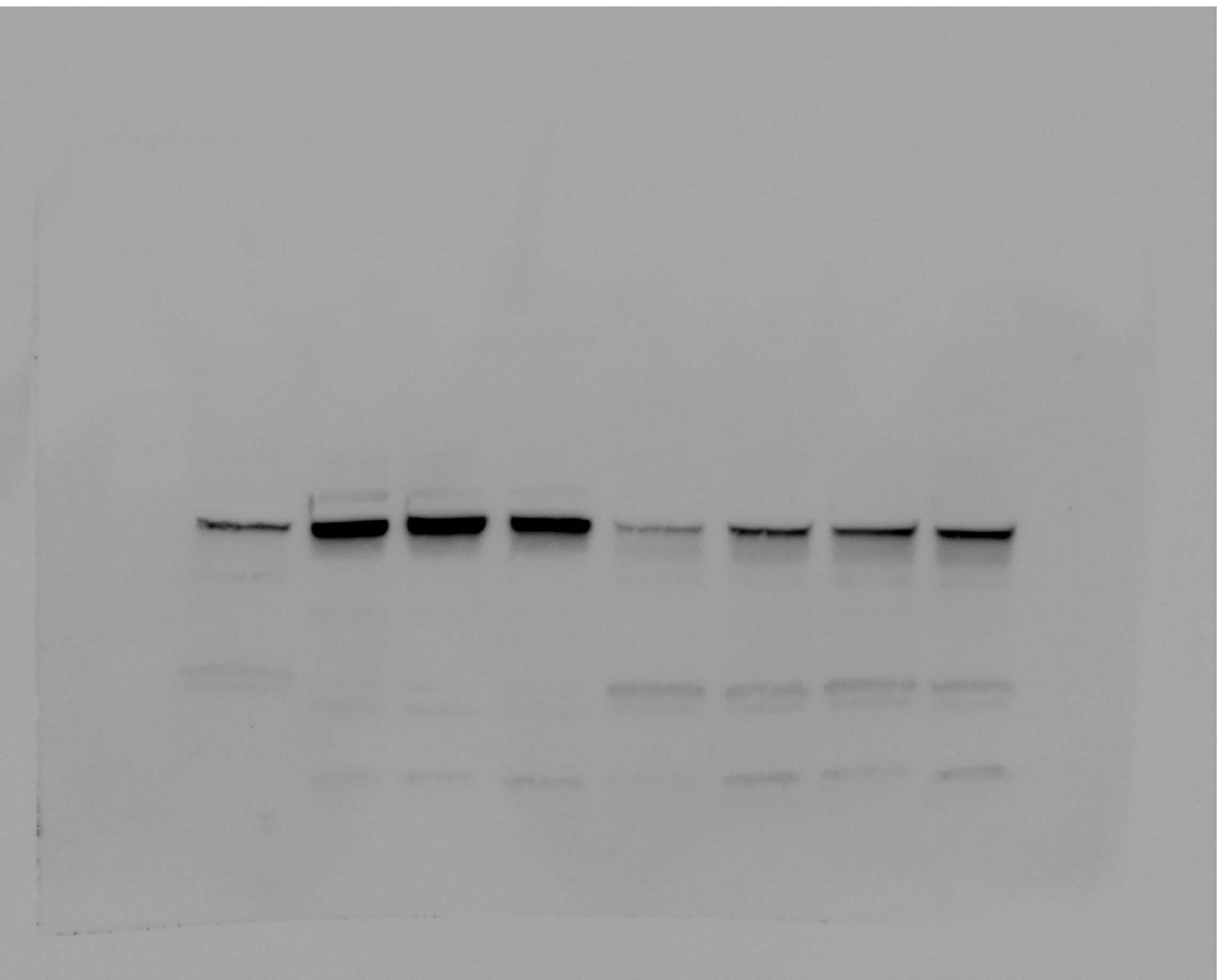

Supplement: Supplementary file 1 [file DataSheet3.zip › figure5-representative-blots/figure5C-PARKIN.tif]

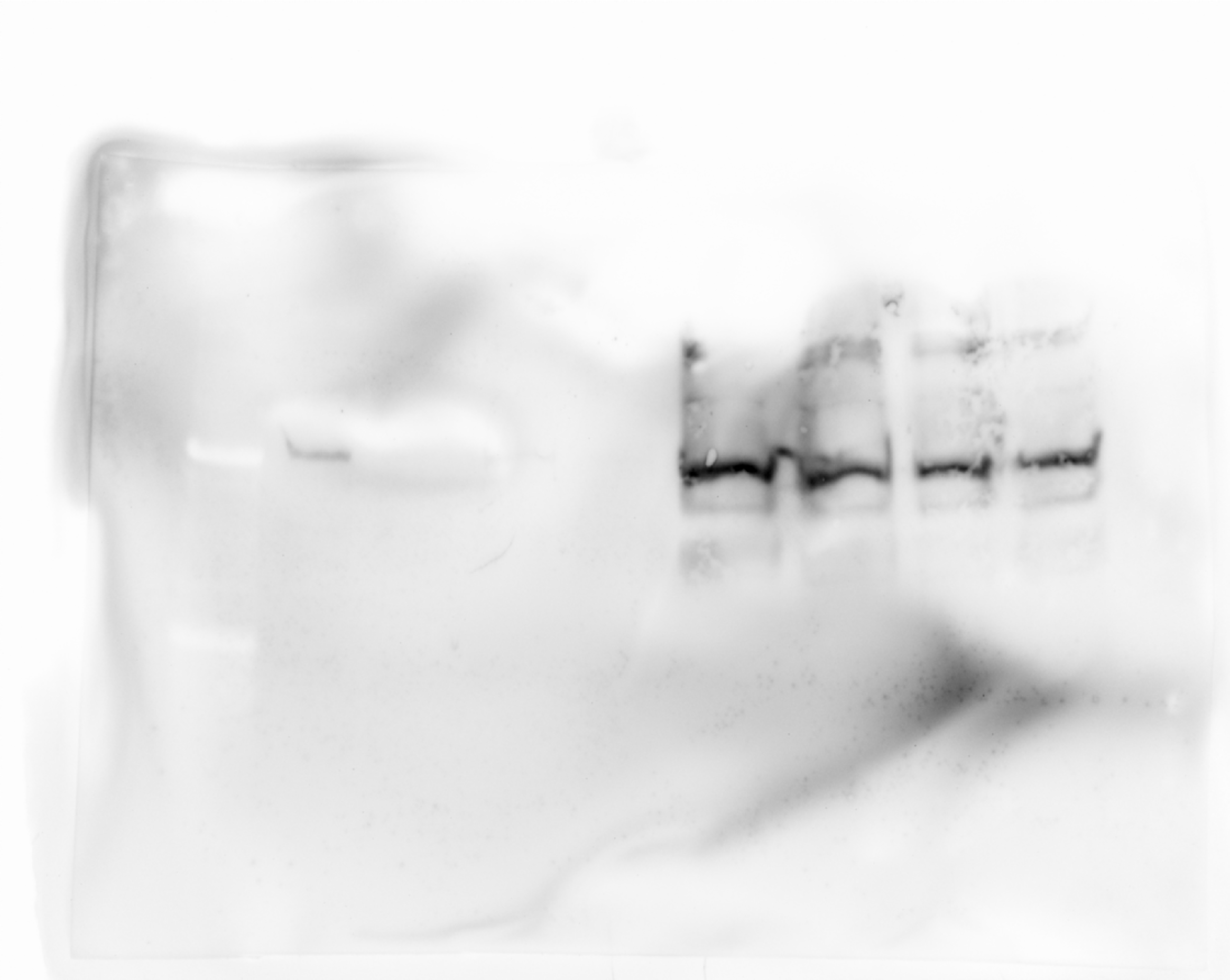

Supplement: Supplementary file 1 [file DataSheet3.zip › figure5-representative-blots/figure5A-DRP1.tif]

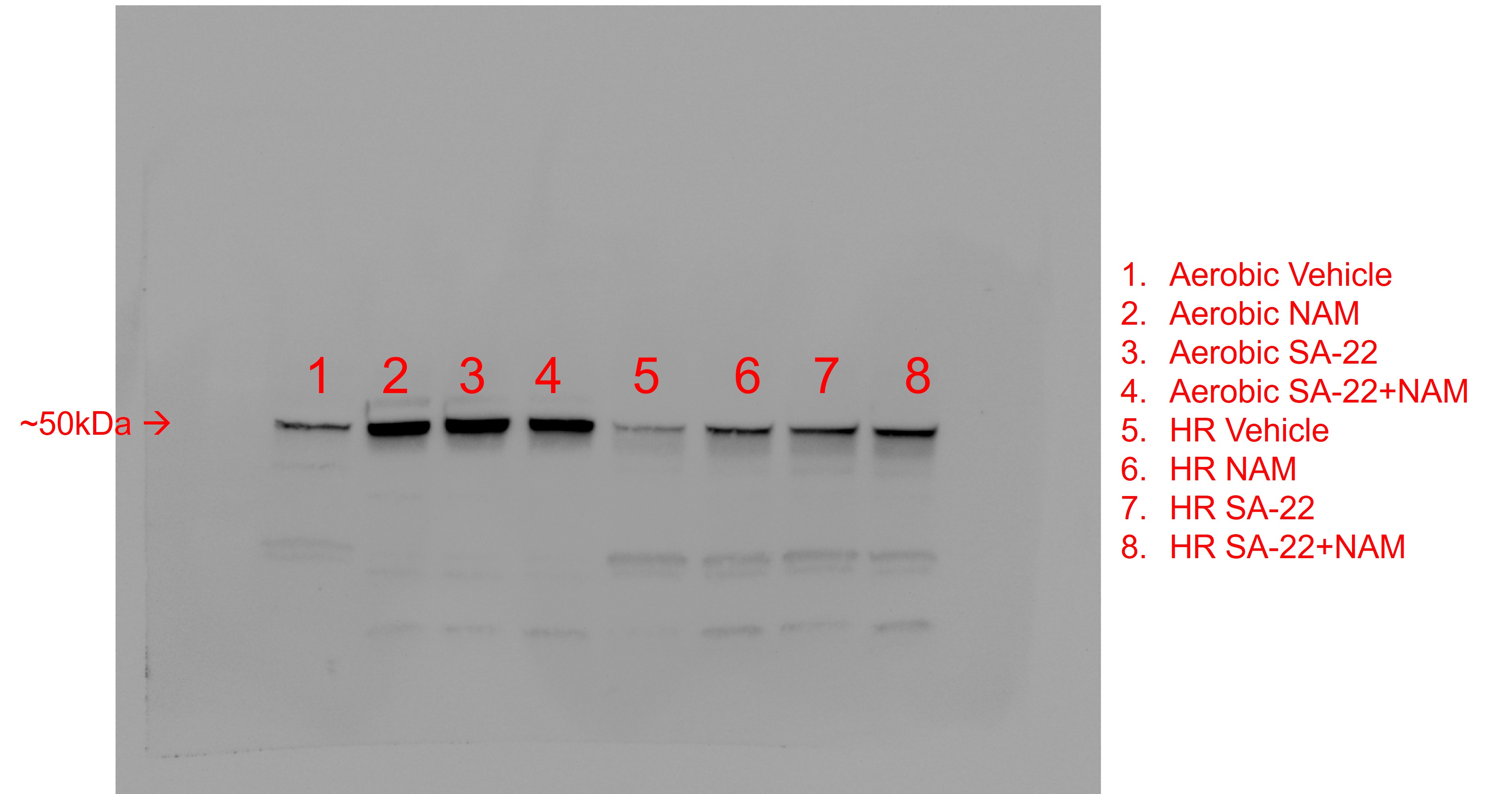

Supplement: Supplementary file 1 [file DataSheet3.zip › figure5-representative-blots/figure5C-PARKIN-labels.jpg]

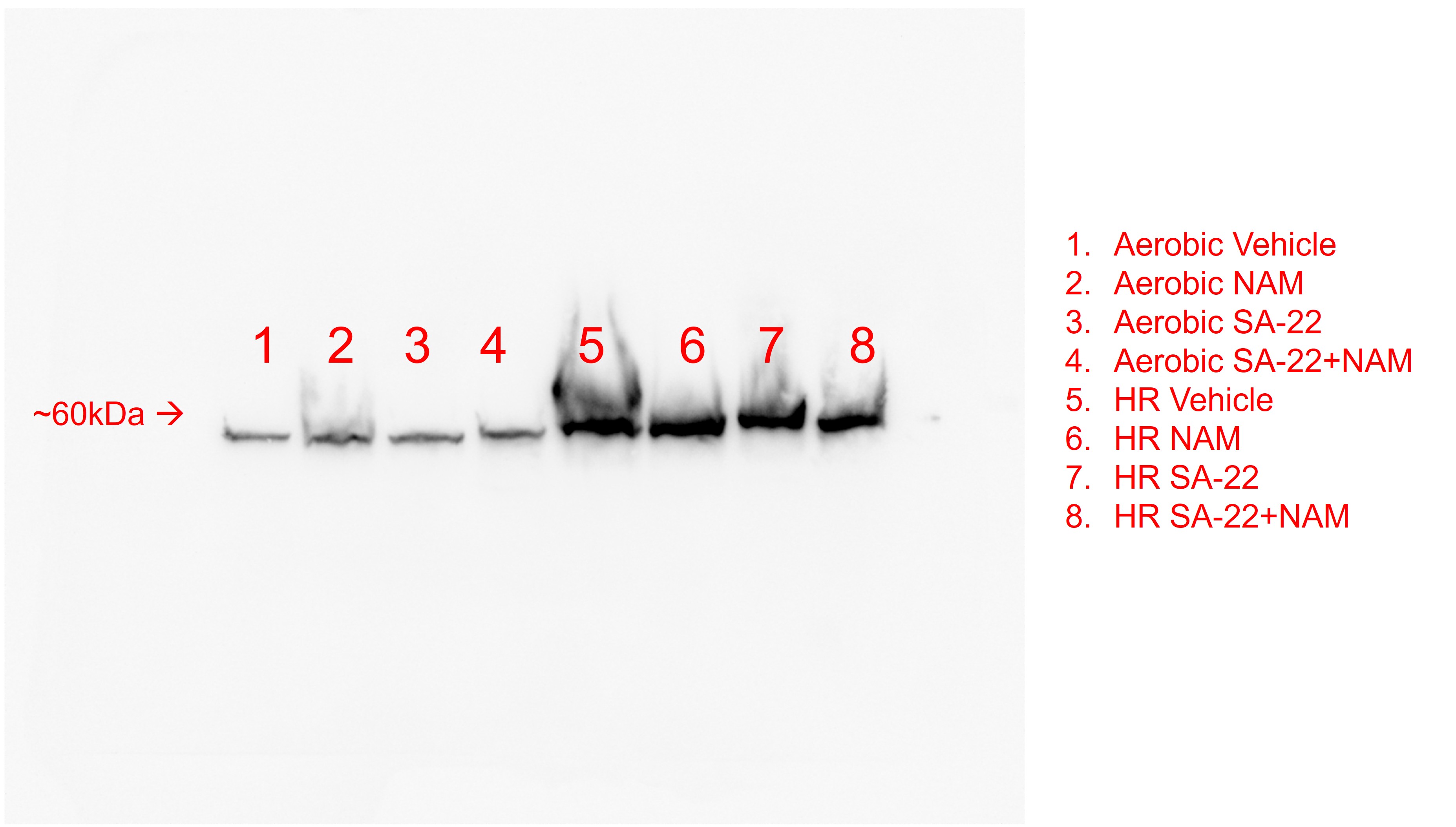

Supplement: Supplementary file 1 [file DataSheet3.zip › figure5-representative-blots/figure5-HSP60-labels.jpg]

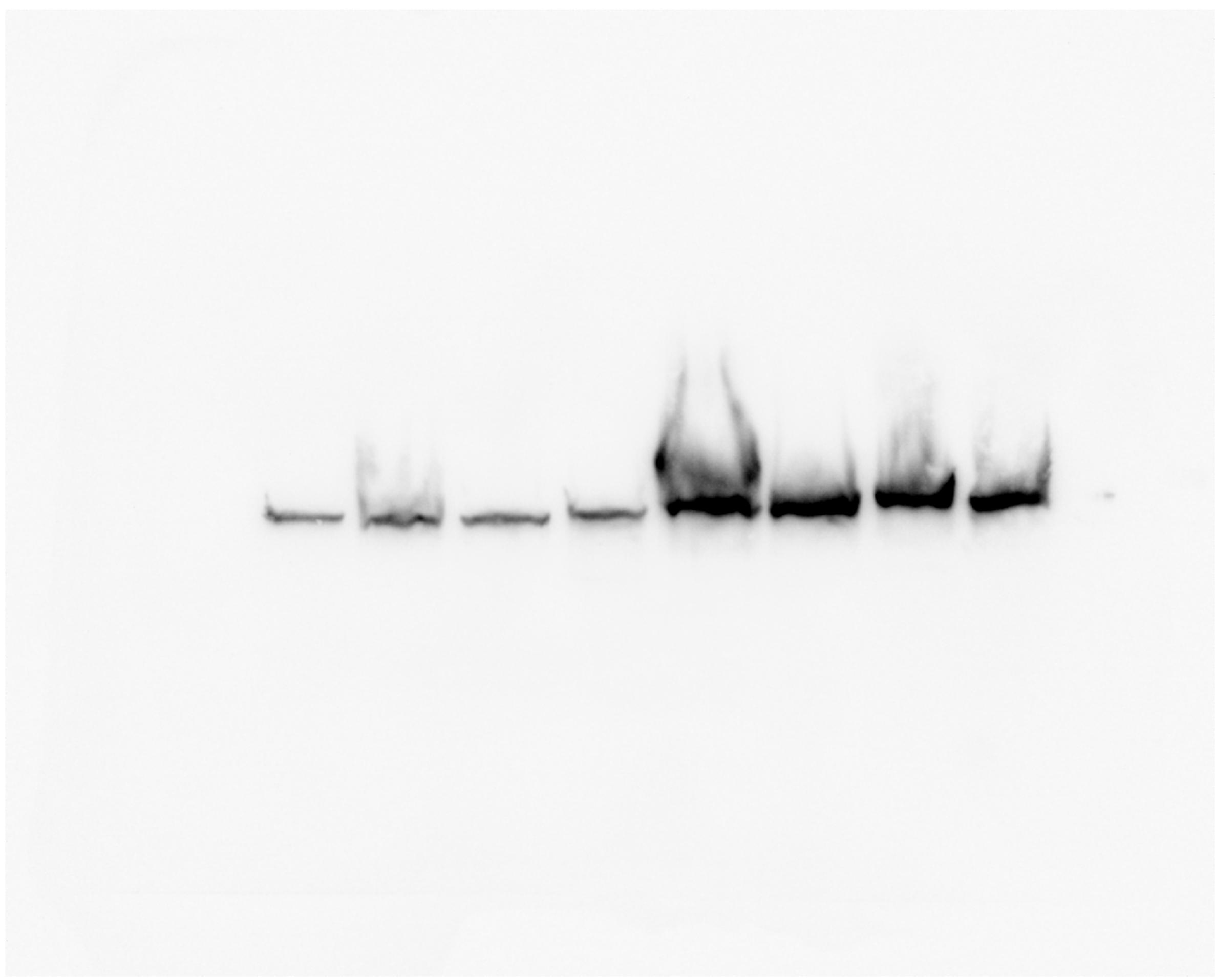

Supplement: Supplementary file 1 [file DataSheet3.zip › figure5-representative-blots/figure5-HSP60.tif]

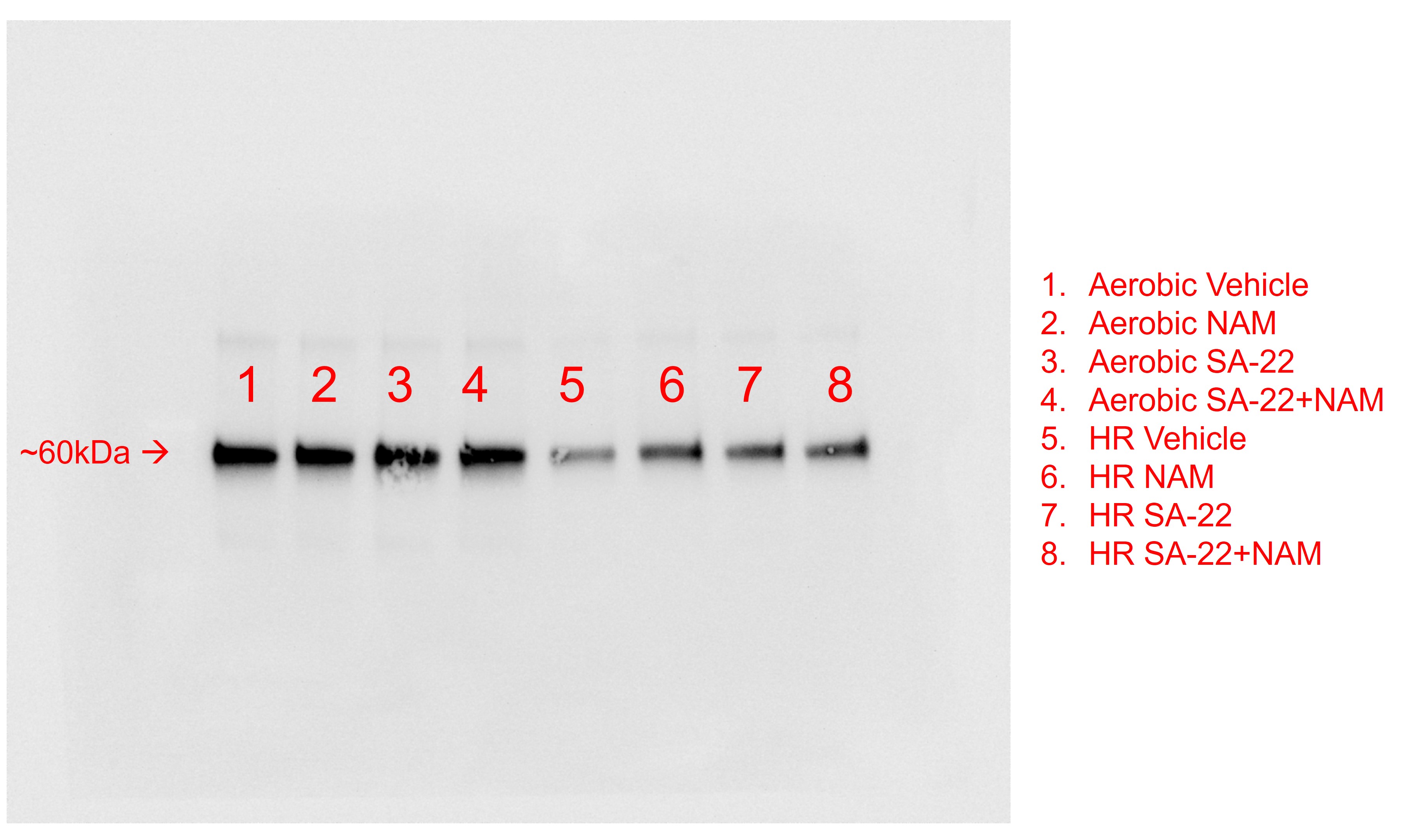

Supplement: Supplementary file 1 [file DataSheet3.zip › figure5-representative-blots/figure5D-P62-labels.jpg]

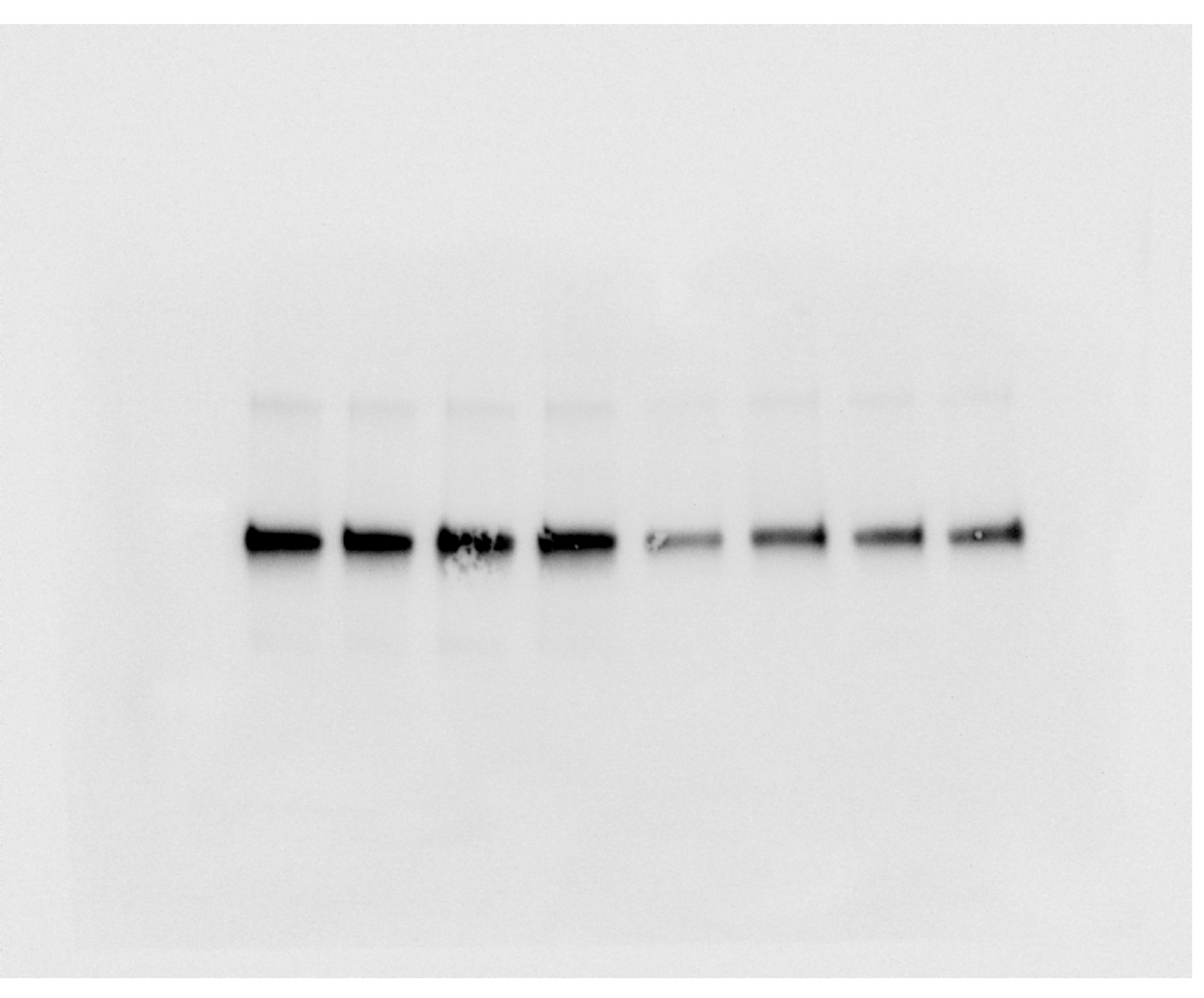

Supplement: Supplementary file 1 [file DataSheet3.zip › figure5-representative-blots/figure5D-P62.tif]

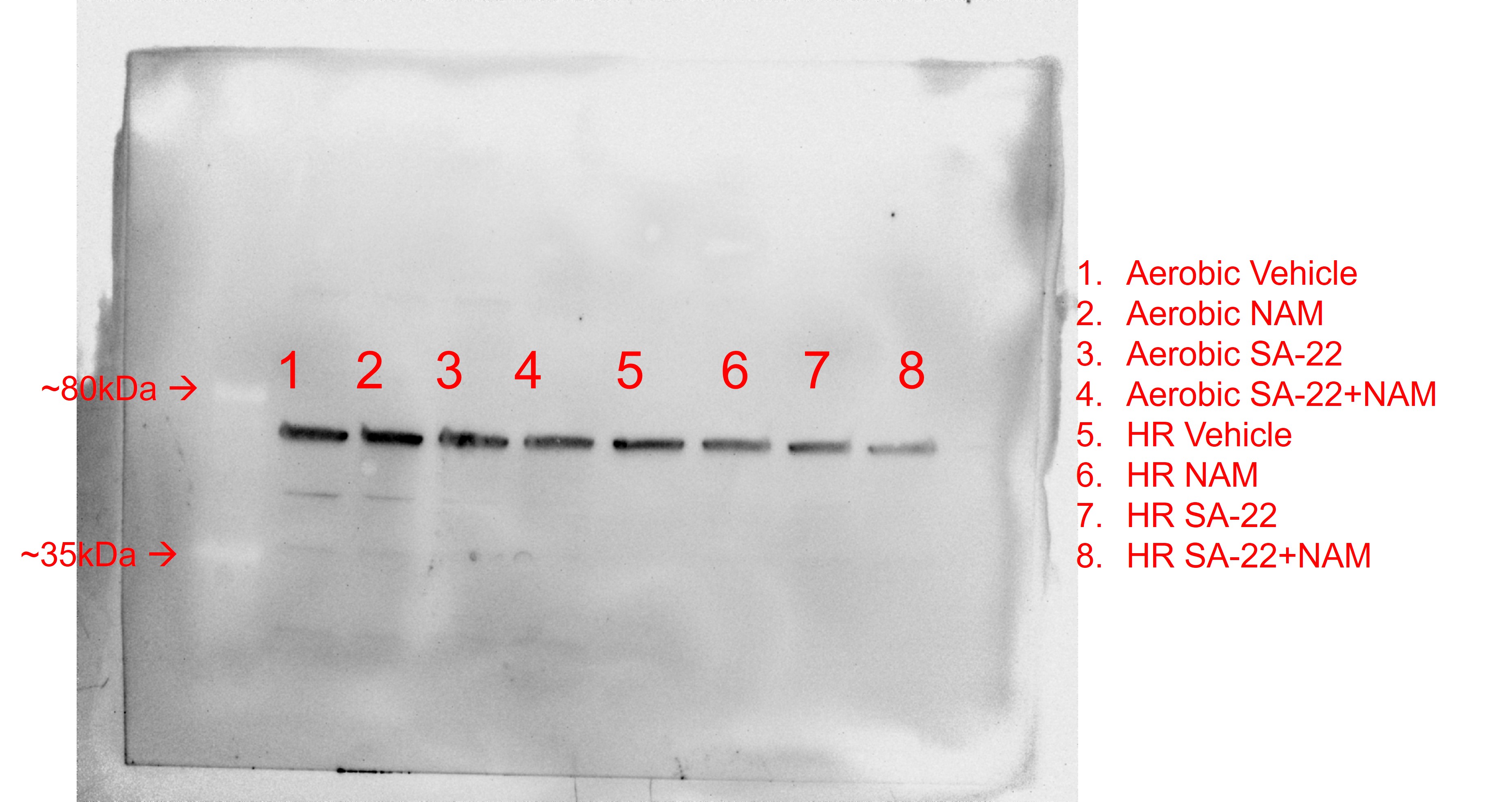

Supplement: Supplementary file 1 [file DataSheet3.zip › figure5-representative-blots/figure5B-PINK1-labels.jpg]

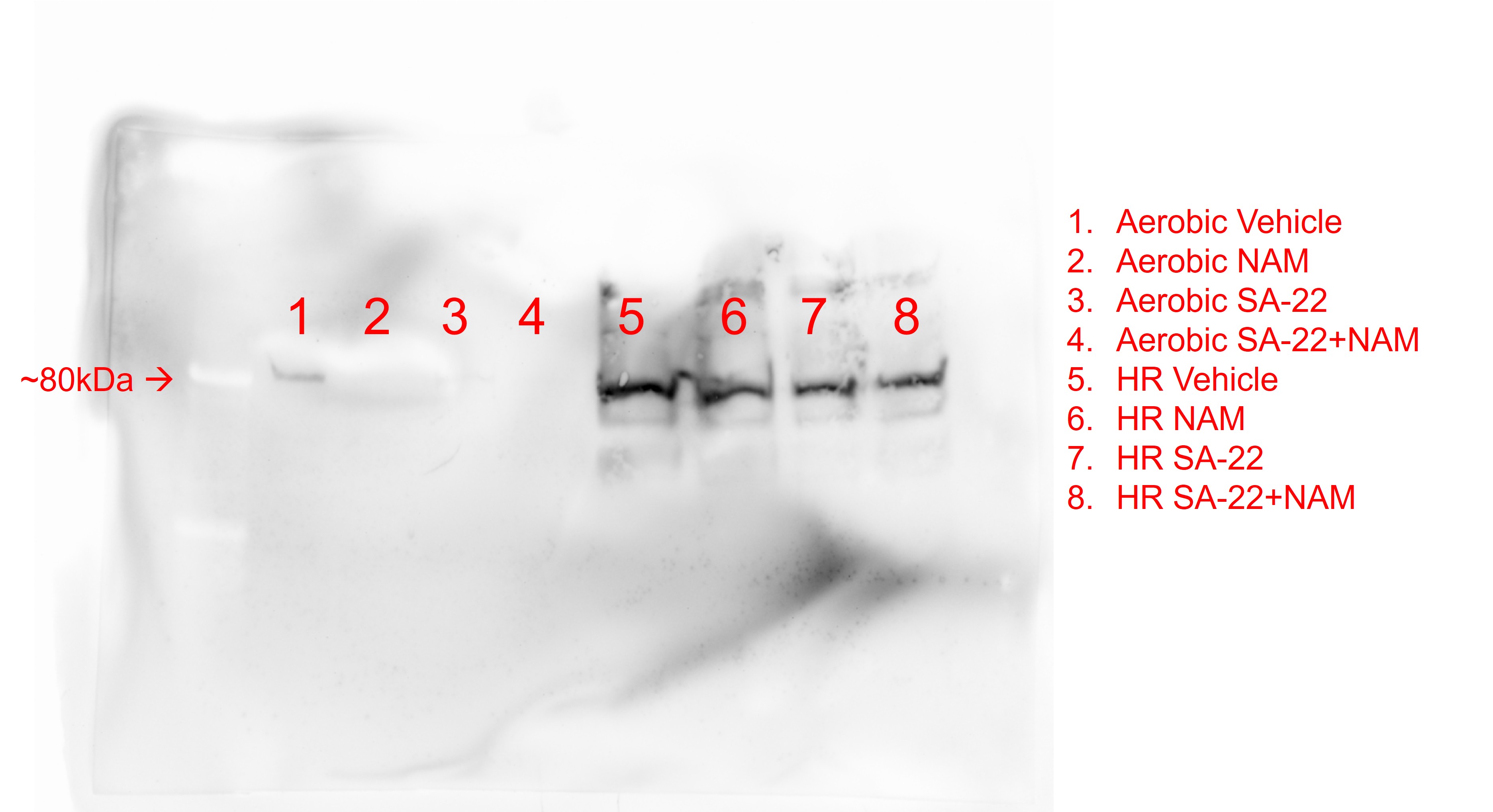

Supplement: Supplementary file 1 [file DataSheet3.zip › figure5-representative-blots/figure5A-DRP1-labels(1).jpg]

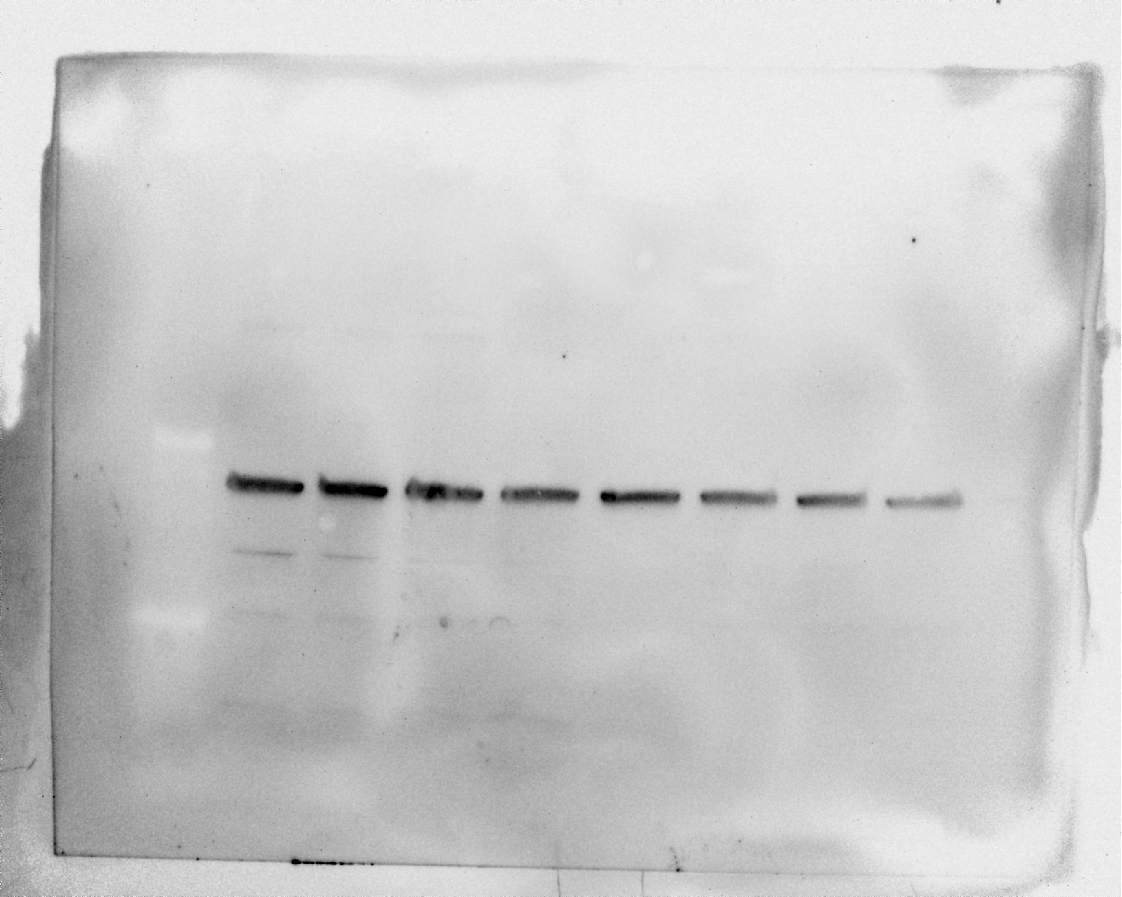

Supplement: Supplementary file 1 [file DataSheet3.zip › figure5-representative-blots/figure5B-PINK1.jpg]

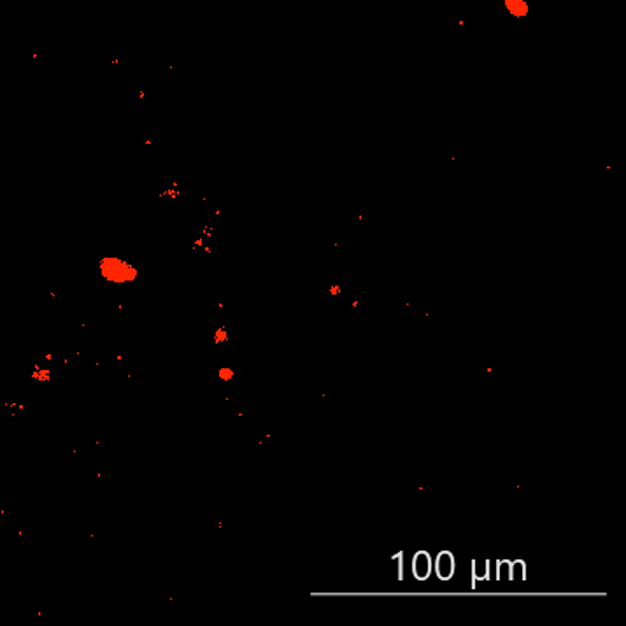

Supplement: Supplementary file 3 [file DataSheet1.zip › figure4-fluorescence-images/hypoxic-vehicle-red.tif]

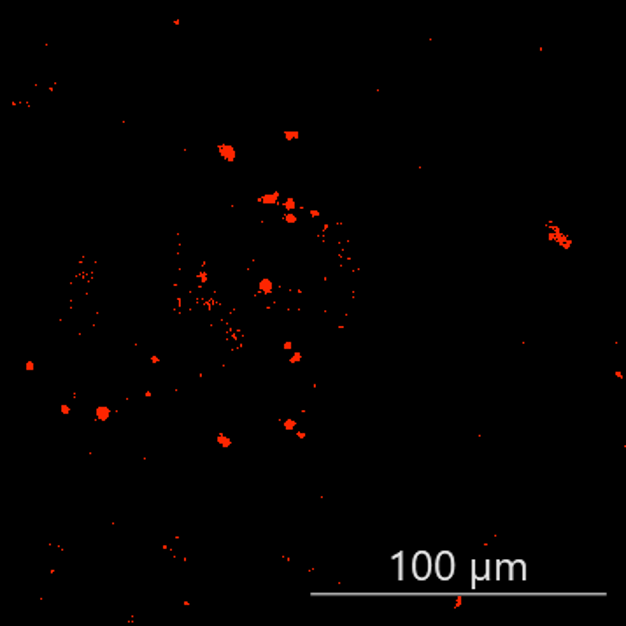

Supplement: Supplementary file 3 [file DataSheet1.zip › figure4-fluorescence-images/hypoxic-SA22+3TYP-red.tif]

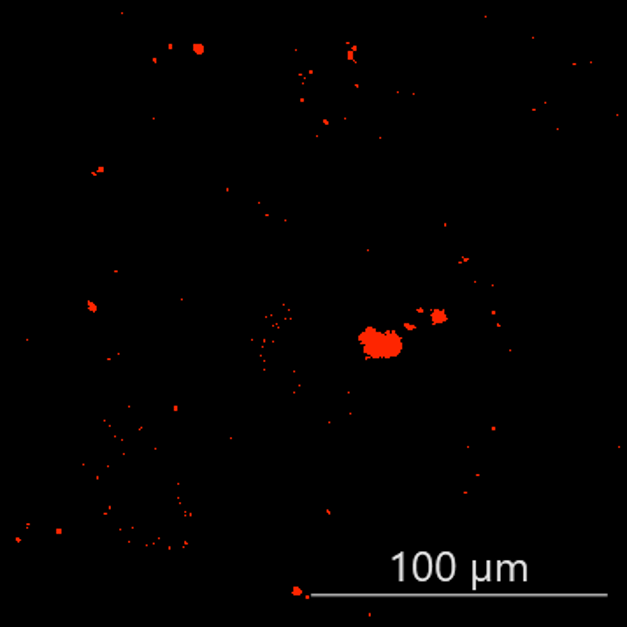

Supplement: Supplementary file 3 [file DataSheet1.zip › figure4-fluorescence-images/hypoxic-EDP+NAM-red.tif]

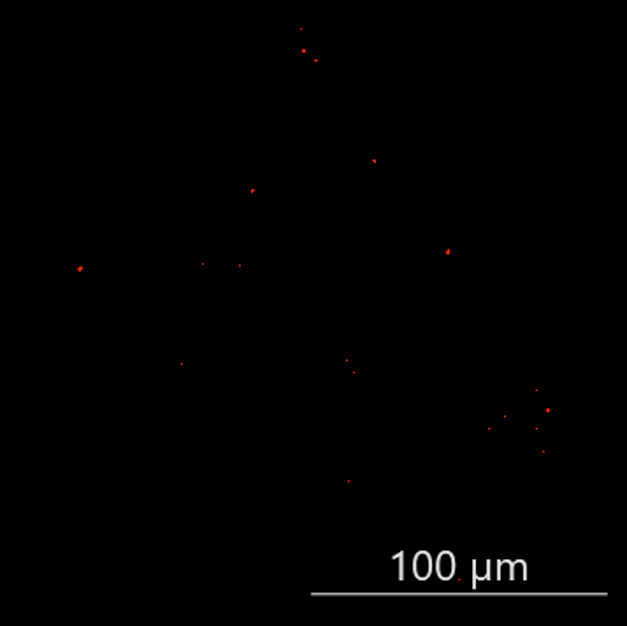

Supplement: Supplementary file 3 [file DataSheet1.zip › figure4-fluorescence-images/hypoxic-SA22-red.tif]

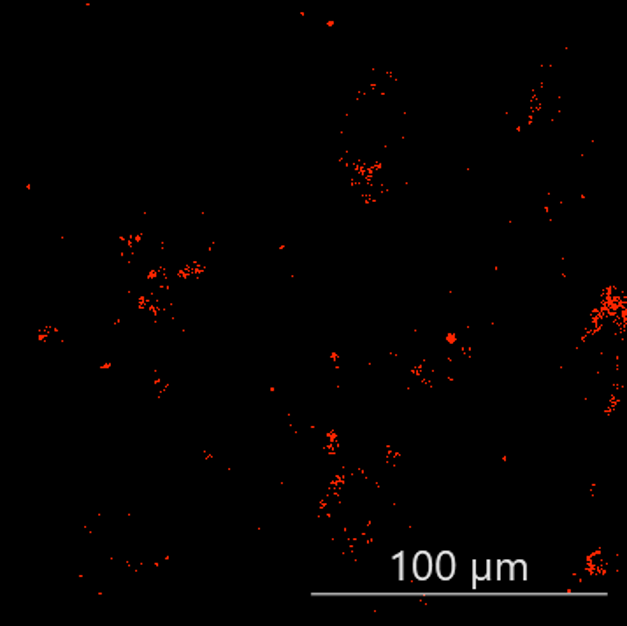

Supplement: Supplementary file 3 [file DataSheet1.zip › figure4-fluorescence-images/hypoxic-FCCP-red.tif]

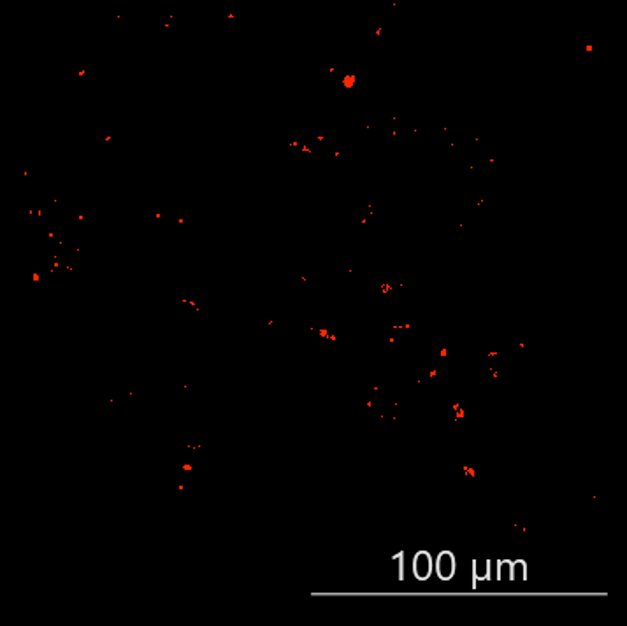

Supplement: Supplementary file 3 [file DataSheet1.zip › figure4-fluorescence-images/hypoxic-SA22+NAM-red.tif]

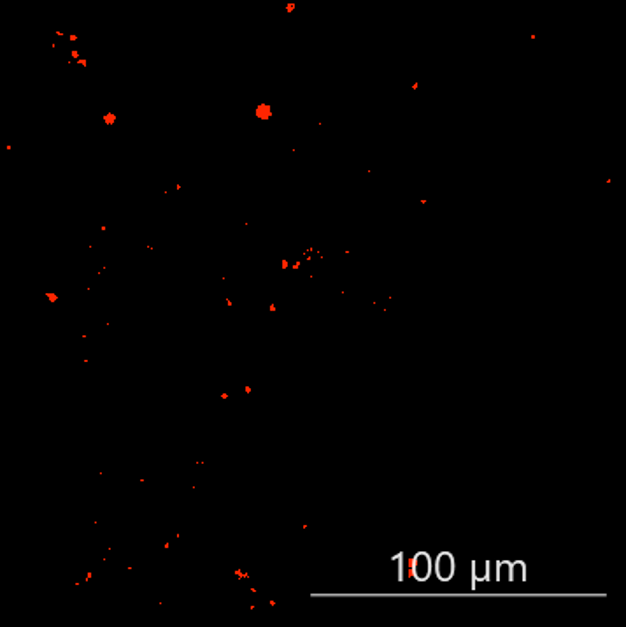

Supplement: Supplementary file 3 [file DataSheet1.zip › figure4-fluorescence-images/hypoxic-EDP+3TYP-red.tif]

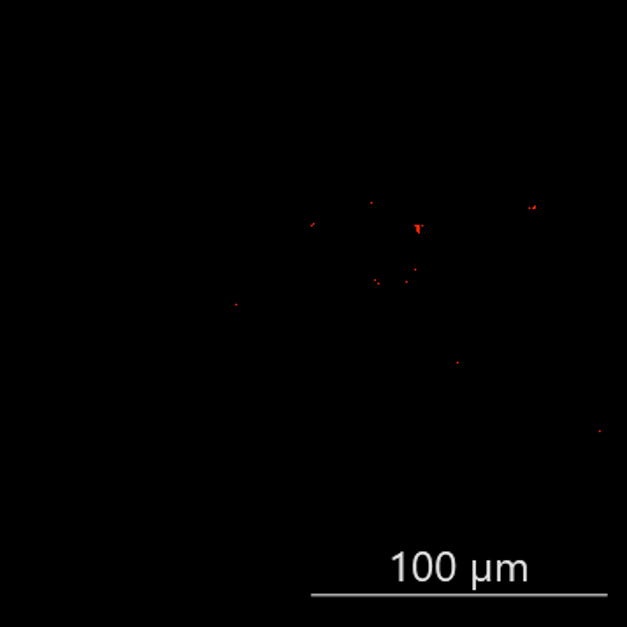

Supplement: Supplementary file 3 [file DataSheet1.zip › figure4-fluorescence-images/aerobic-vehicle-red.tif]

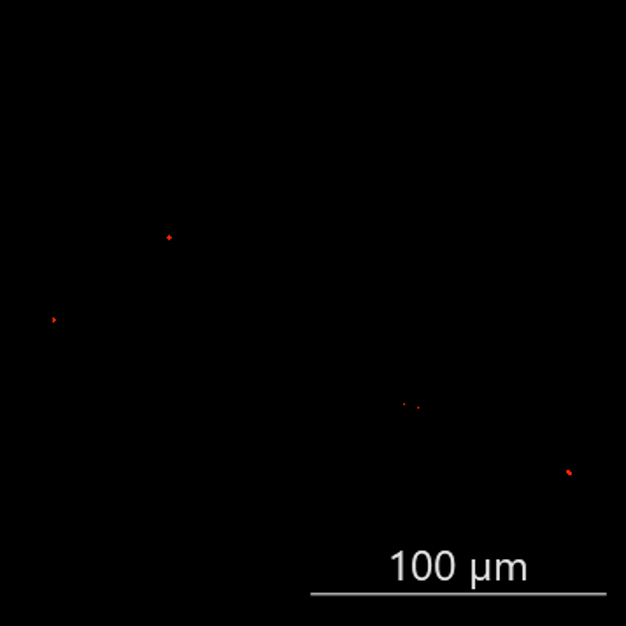

Supplement: Supplementary file 3 [file DataSheet1.zip › figure4-fluorescence-images/hypoxic-EDP-red.tif]

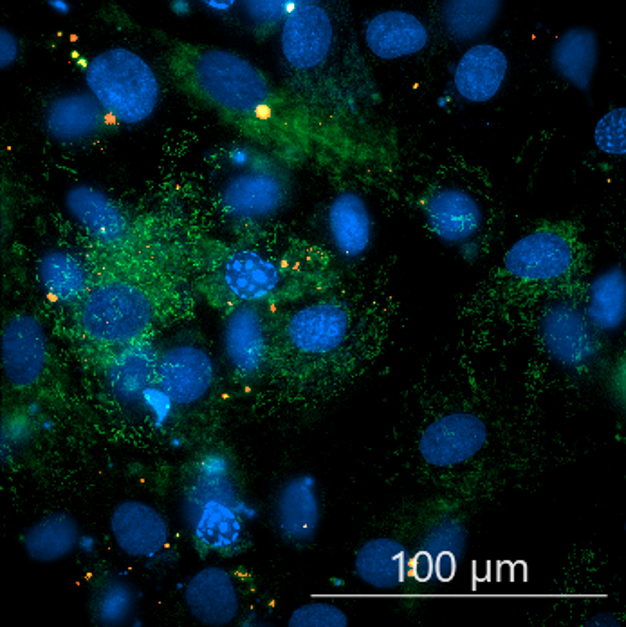

Supplement: Supplementary file 3 [file DataSheet1.zip › figure4-fluorescence-images/hypoxic-EDP+3TYP-merge.tif]

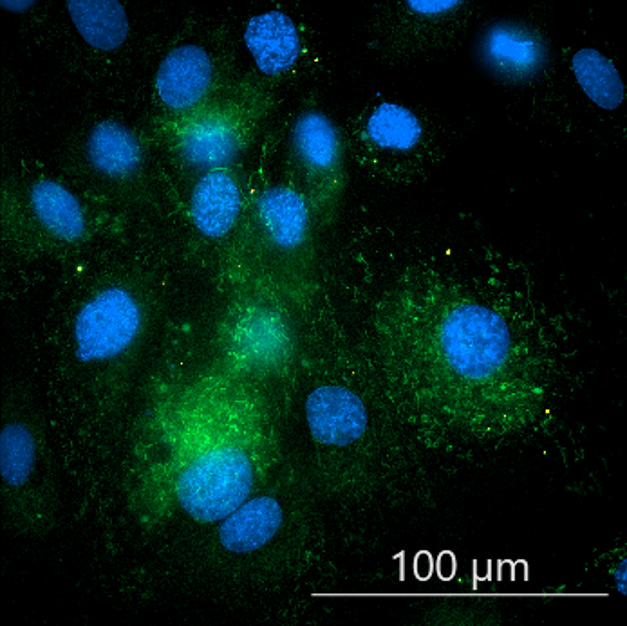

Supplement: Supplementary file 3 [file DataSheet1.zip › figure4-fluorescence-images/hypoxic-SA22-merge.tif]

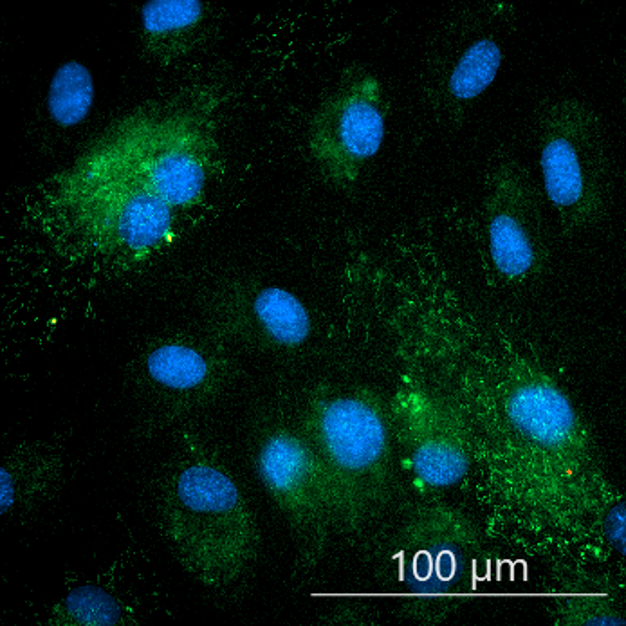

Supplement: Supplementary file 3 [file DataSheet1.zip › figure4-fluorescence-images/hypoxic-EDP-merge.tif]

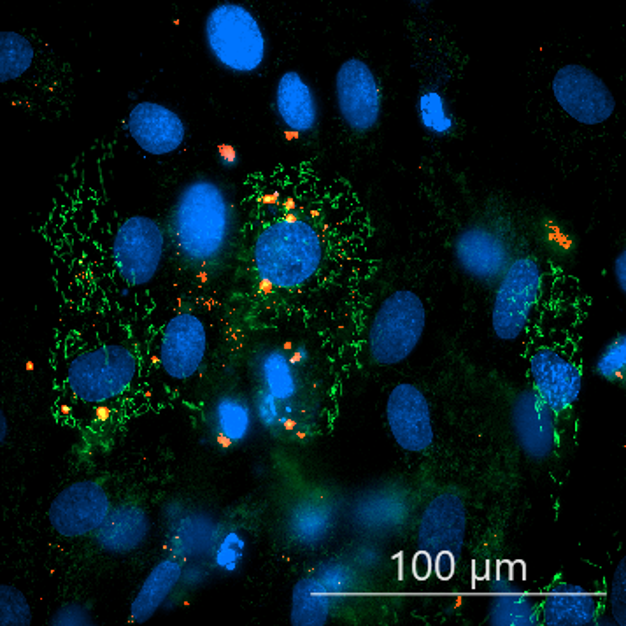

Supplement: Supplementary file 3 [file DataSheet1.zip › figure4-fluorescence-images/hypoxic-SA22+3TYP-merge.tif]

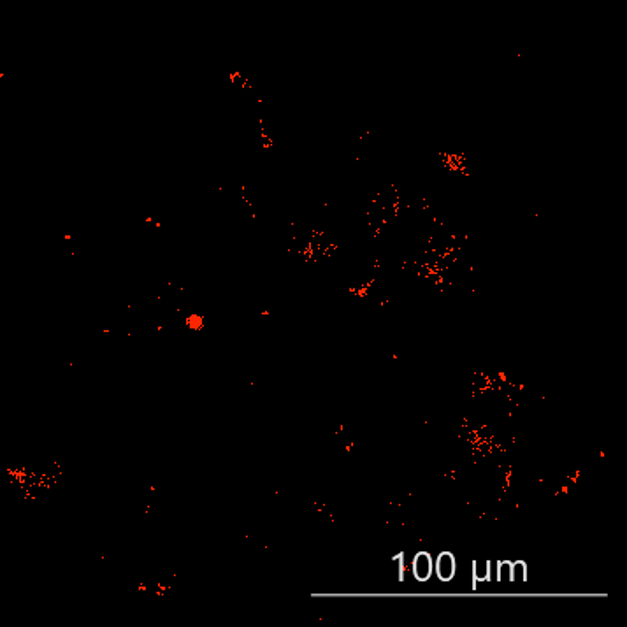

Supplement: Supplementary file 3 [file DataSheet1.zip › figure4-fluorescence-images/aerobic-FCCP-red.tif]

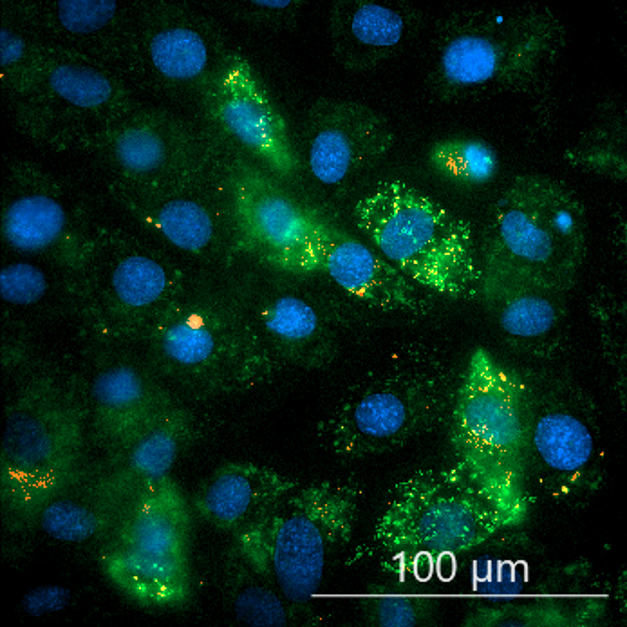

Supplement: Supplementary file 3 [file DataSheet1.zip › figure4-fluorescence-images/aerobic-FCCP-merge.tif]

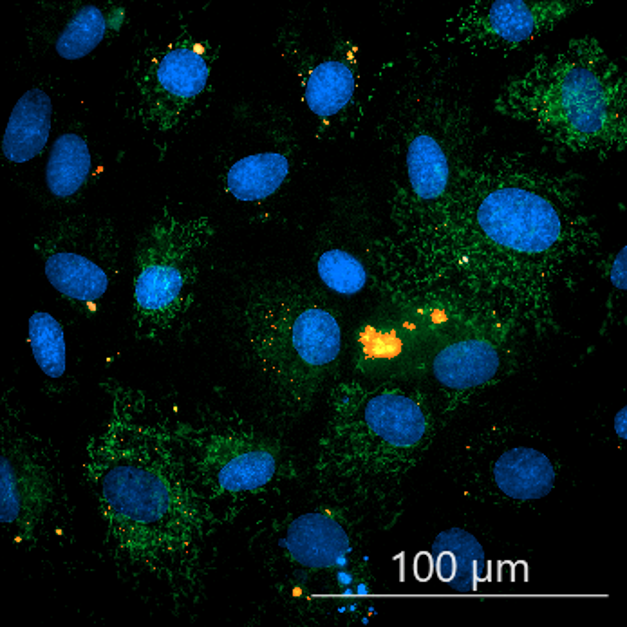

Supplement: Supplementary file 3 [file DataSheet1.zip › figure4-fluorescence-images/hypoxic-EDP+NAM-merge.tif]

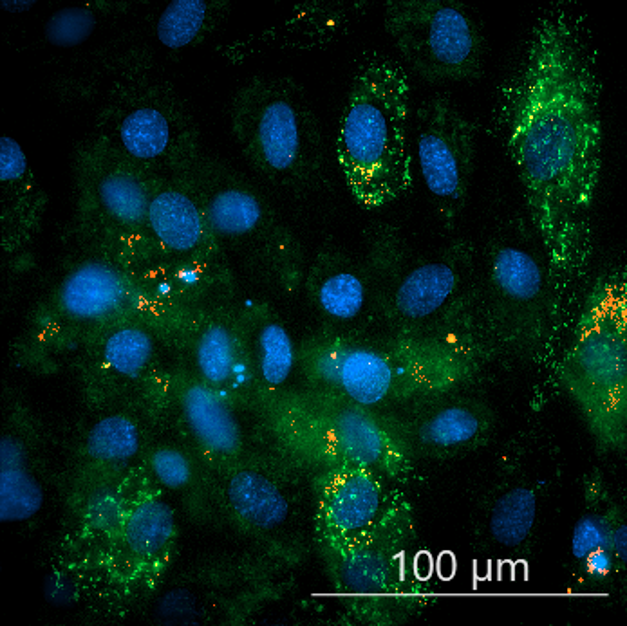

Supplement: Supplementary file 3 [file DataSheet1.zip › figure4-fluorescence-images/hypoxic-FCCP-merge.tif]

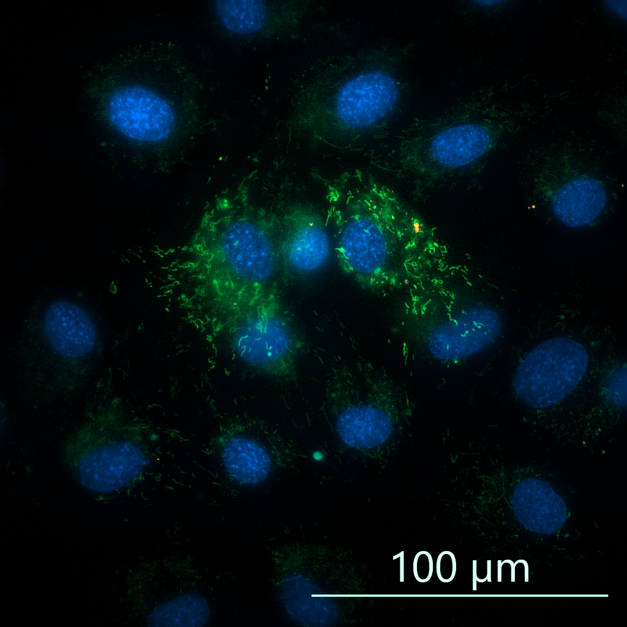

Supplement: Supplementary file 3 [file DataSheet1.zip › figure4-fluorescence-images/aerobic-vehicle-merge.tif]

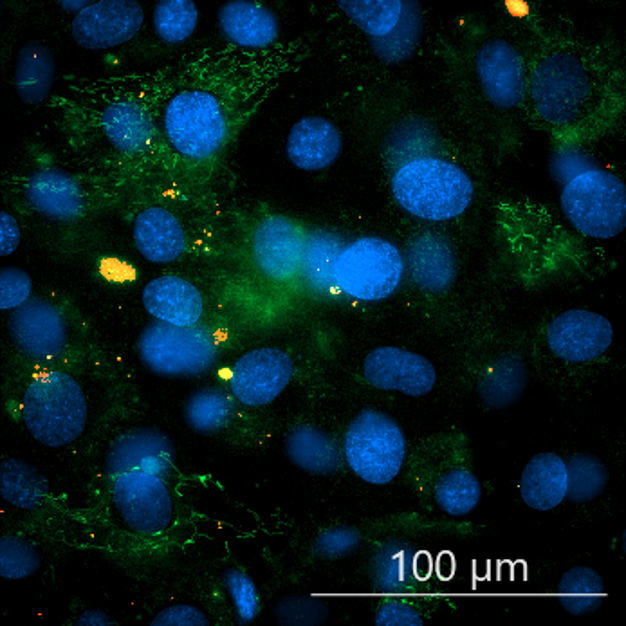

Supplement: Supplementary file 3 [file DataSheet1.zip › figure4-fluorescence-images/hypoxic-vehicle-merge.tif]

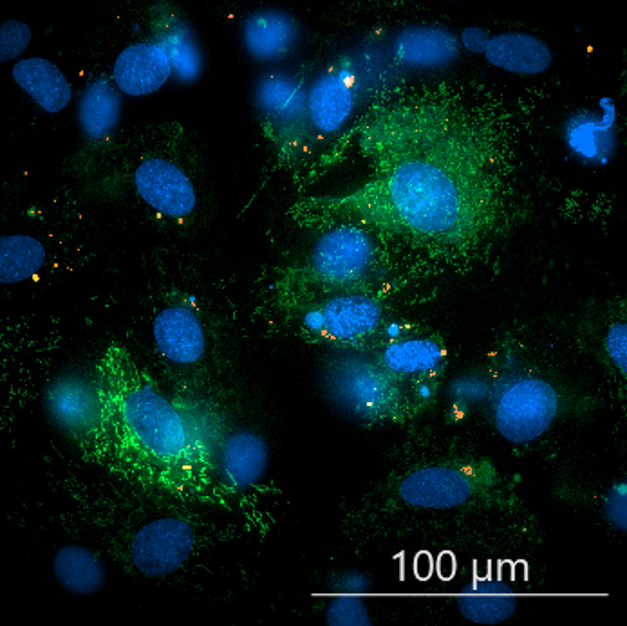

Supplement: Supplementary file 3 [file DataSheet1.zip › figure4-fluorescence-images/hypoxic-SA22+NAM-merge.tif]

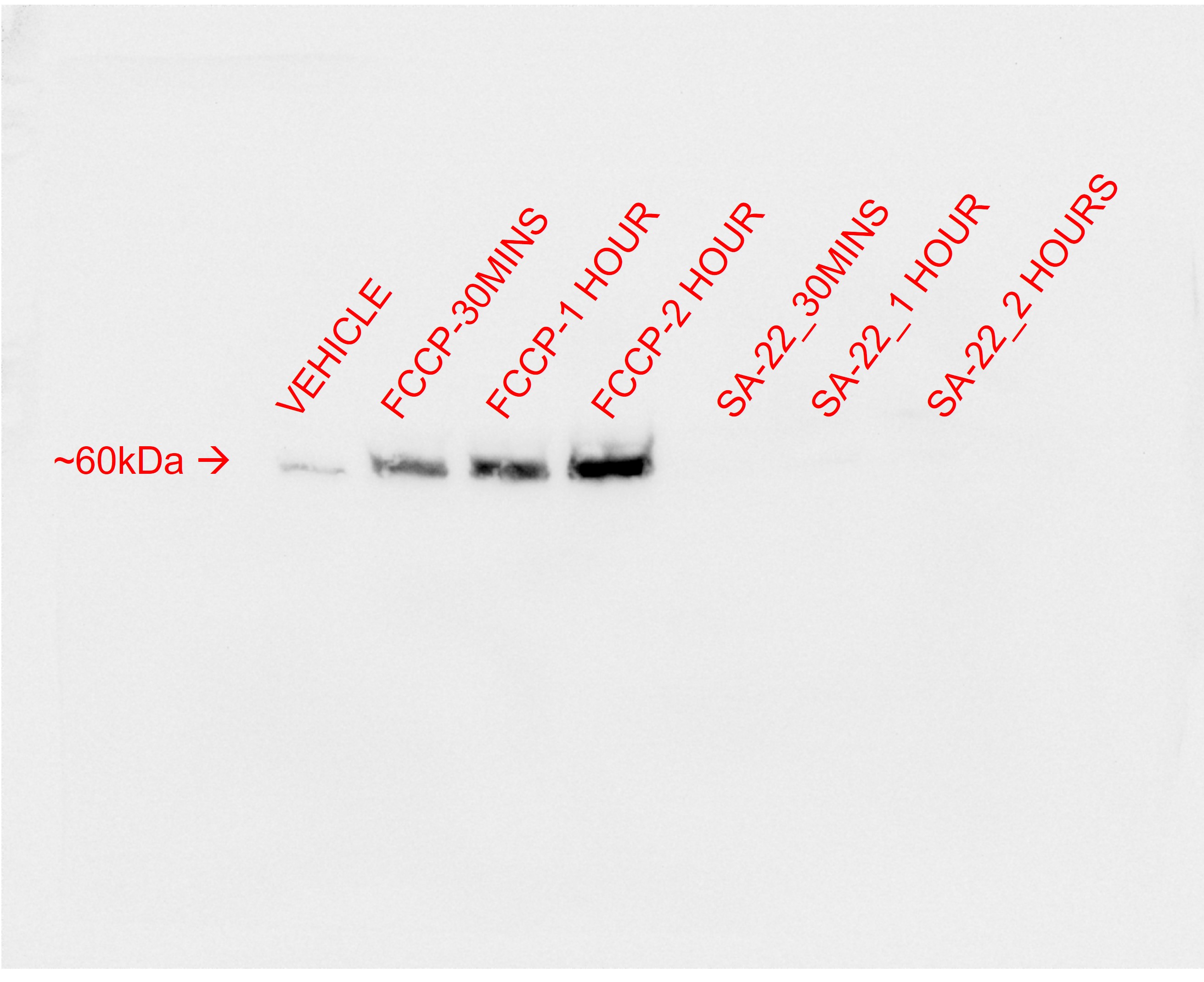

Supplement: Supplementary file 4 [file DataSheet2.zip › figure6-representative-blots/figure6D-P62-labels.jpg]

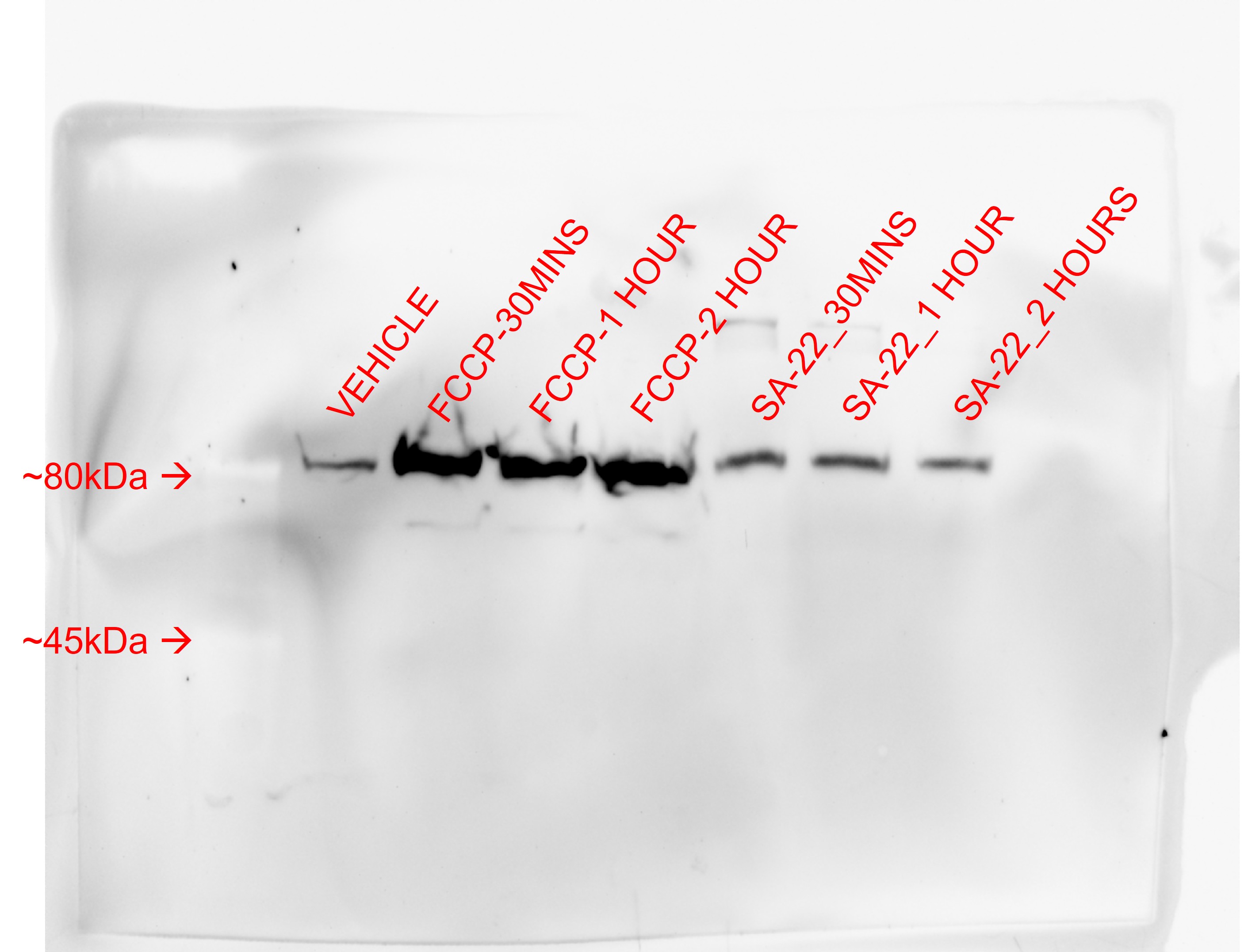

Supplement: Supplementary file 4 [file DataSheet2.zip › figure6-representative-blots/figure6A-DRP1-labels.jpg]

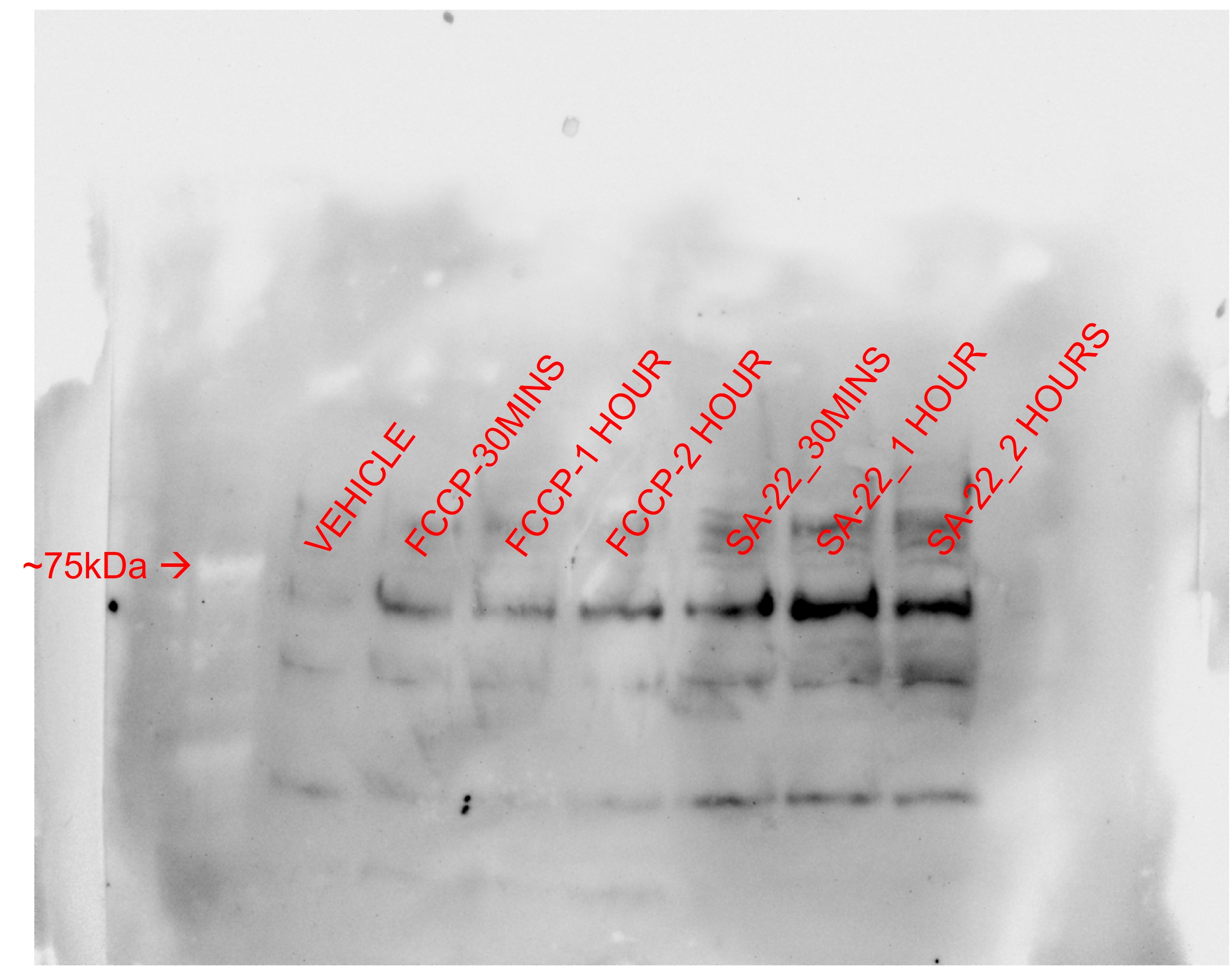

Supplement: Supplementary file 4 [file DataSheet2.zip › figure6-representative-blots/figure6C-PARKIN-labels.jpg]

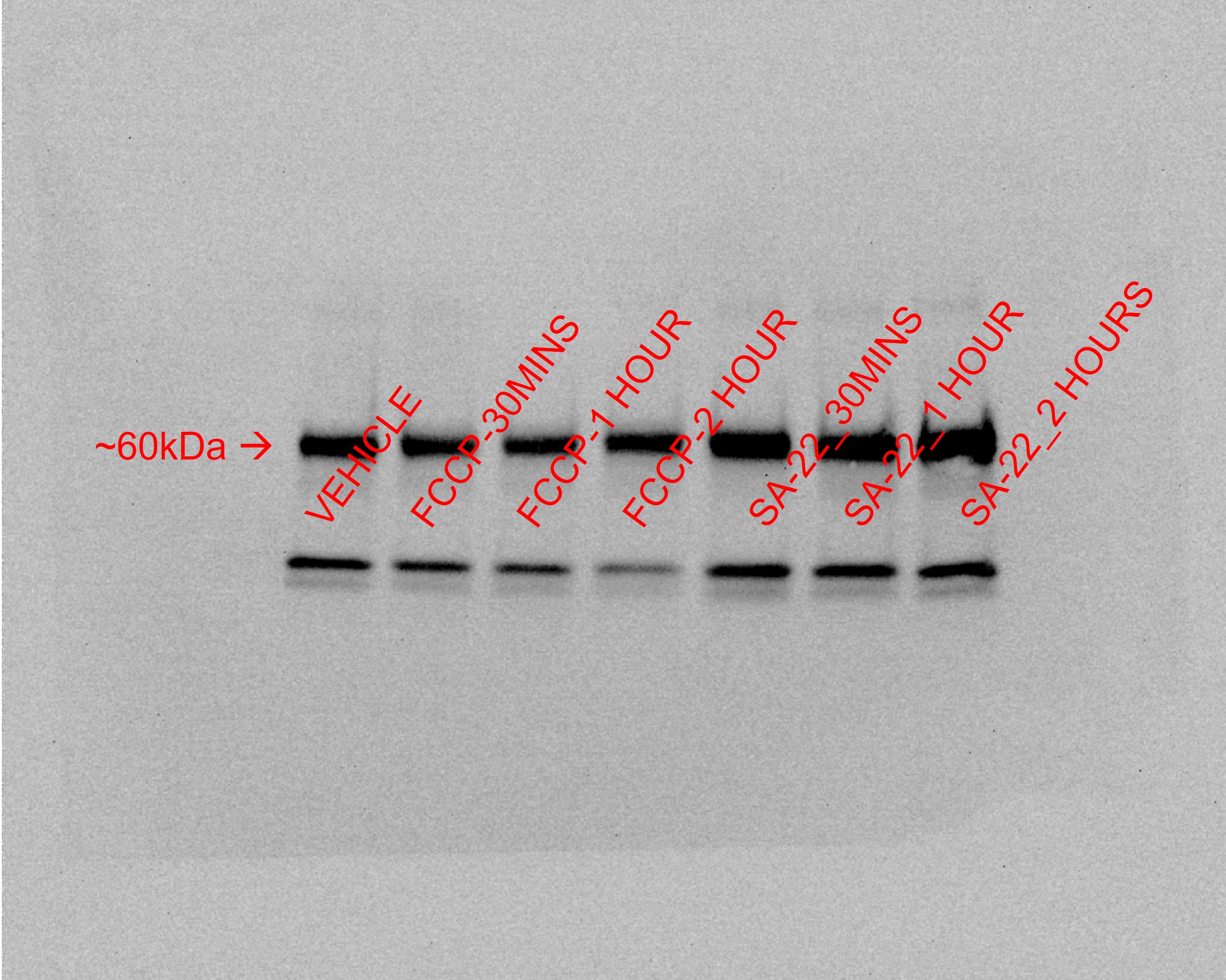

Supplement: Supplementary file 4 [file DataSheet2.zip › figure6-representative-blots/figure6-VDAC-labels.jpg]

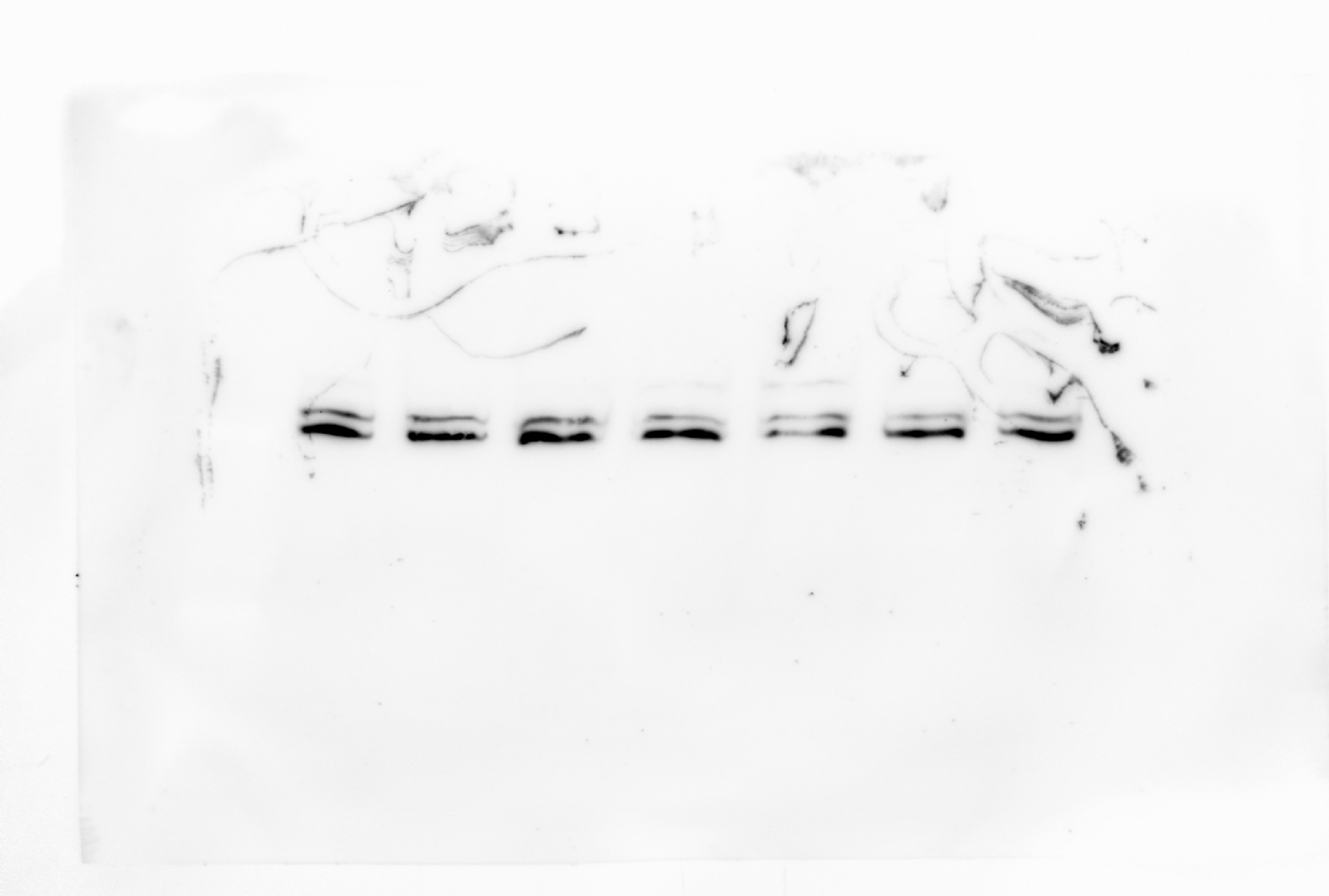

Supplement: Supplementary file 4 [file DataSheet2.zip › figure6-representative-blots/figure6B-PINK1.tif]

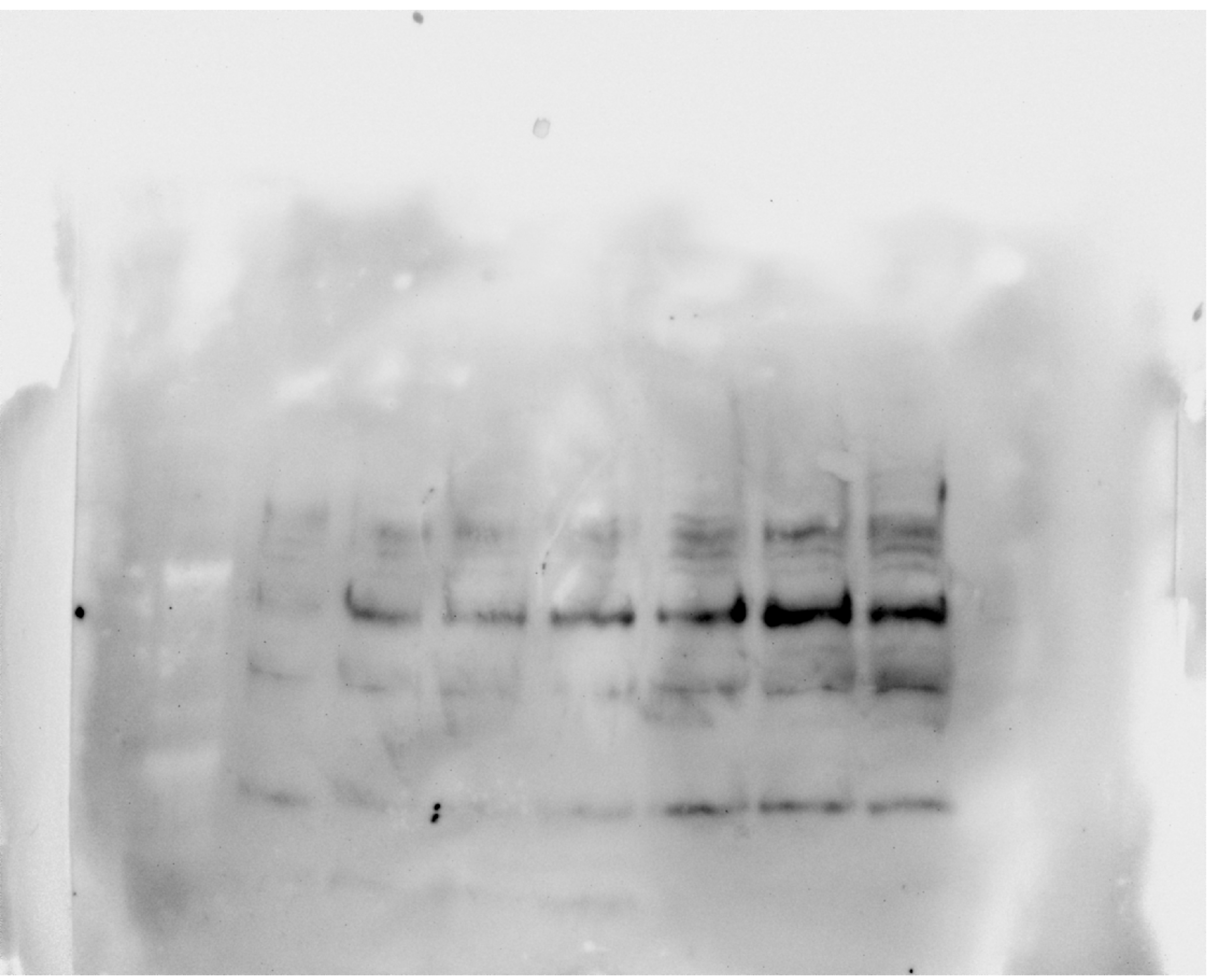

Supplement: Supplementary file 4 [file DataSheet2.zip › figure6-representative-blots/figure6C-PARKIN.tif]

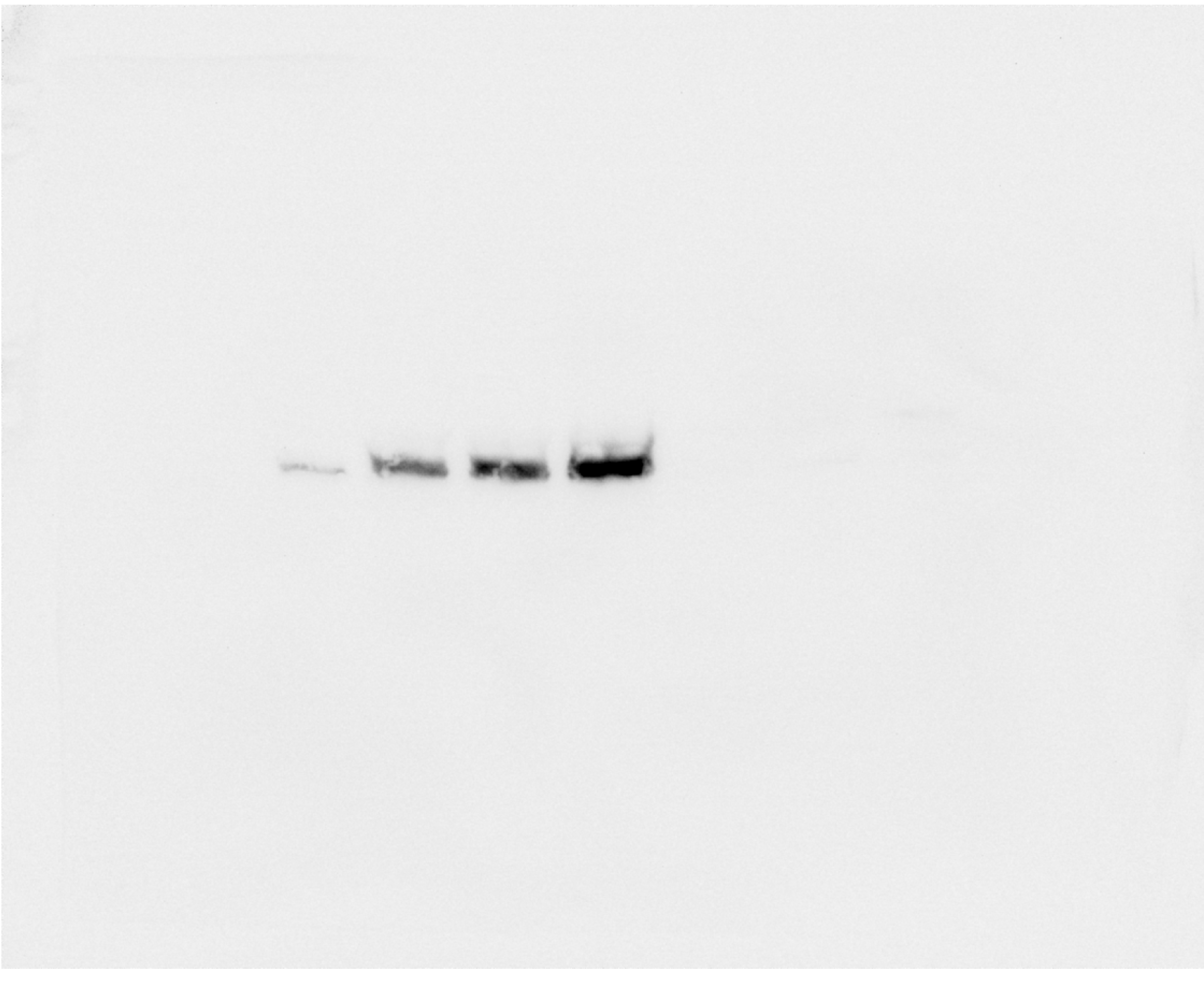

Supplement: Supplementary file 4 [file DataSheet2.zip › figure6-representative-blots/figure6D-P62.tif]

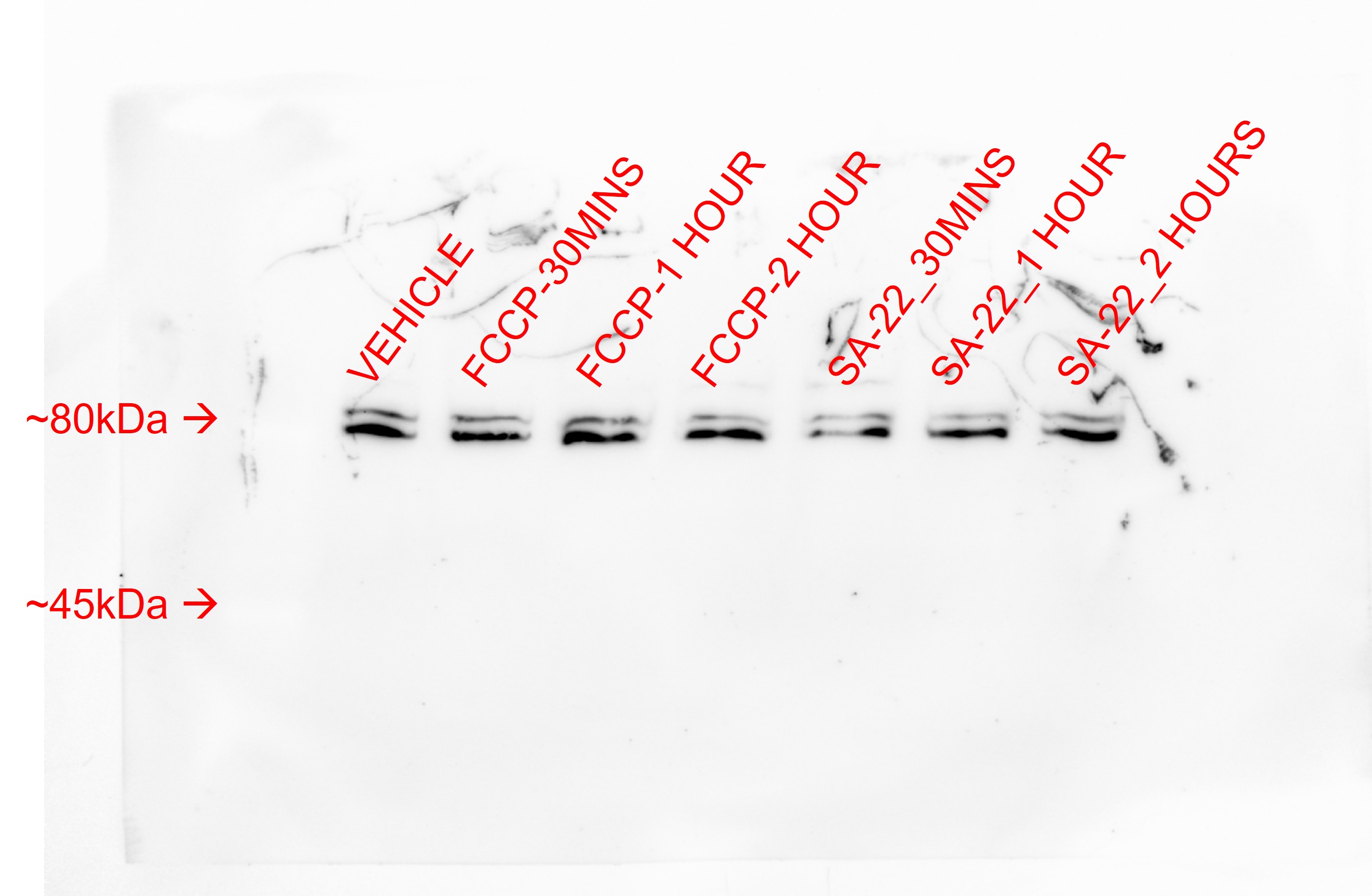

Supplement: Supplementary file 4 [file DataSheet2.zip › figure6-representative-blots/figure6B-PINK1-labels.jpg]

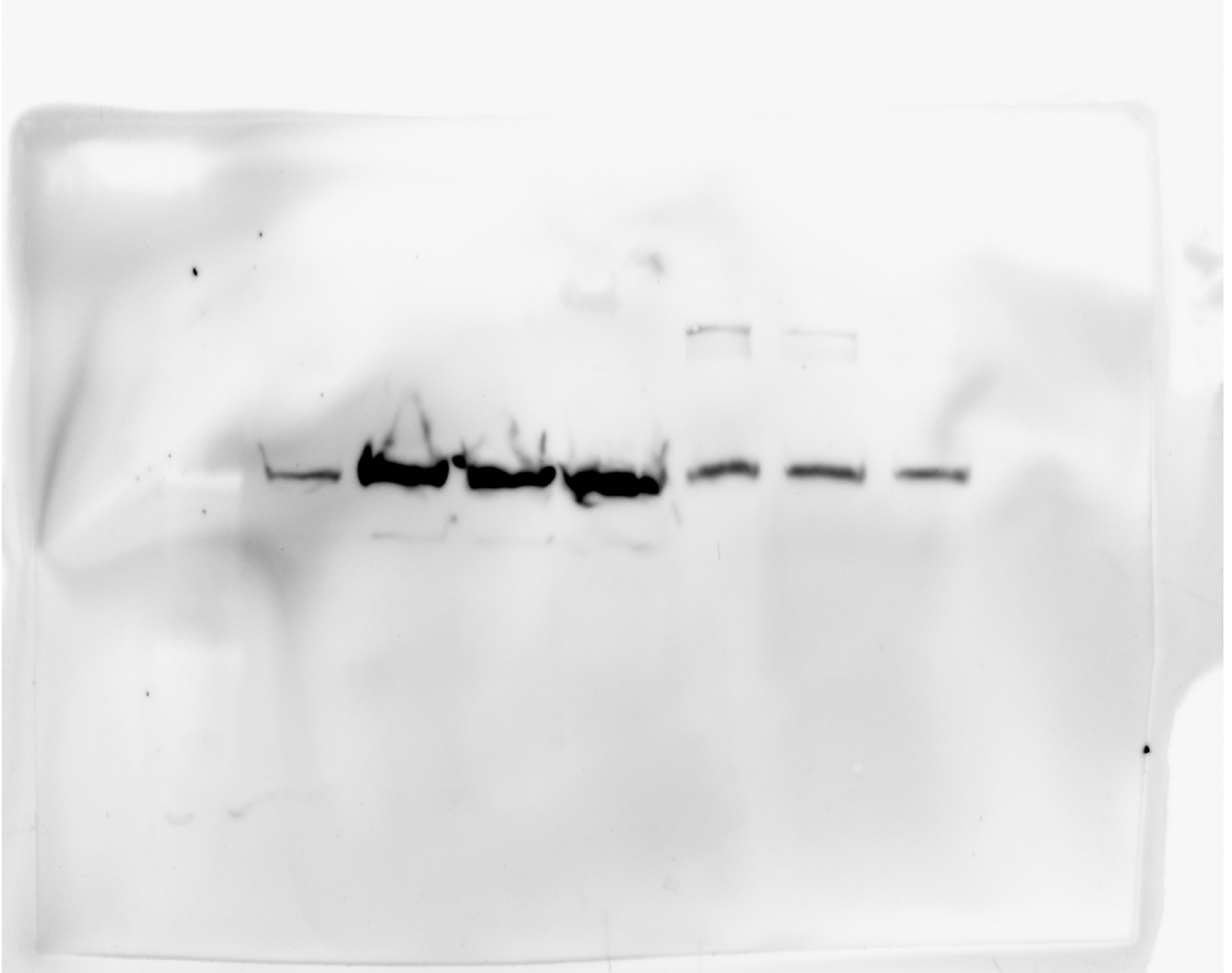

Supplement: Supplementary file 4 [file DataSheet2.zip › figure6-representative-blots/figure6A-DRP1.tif]

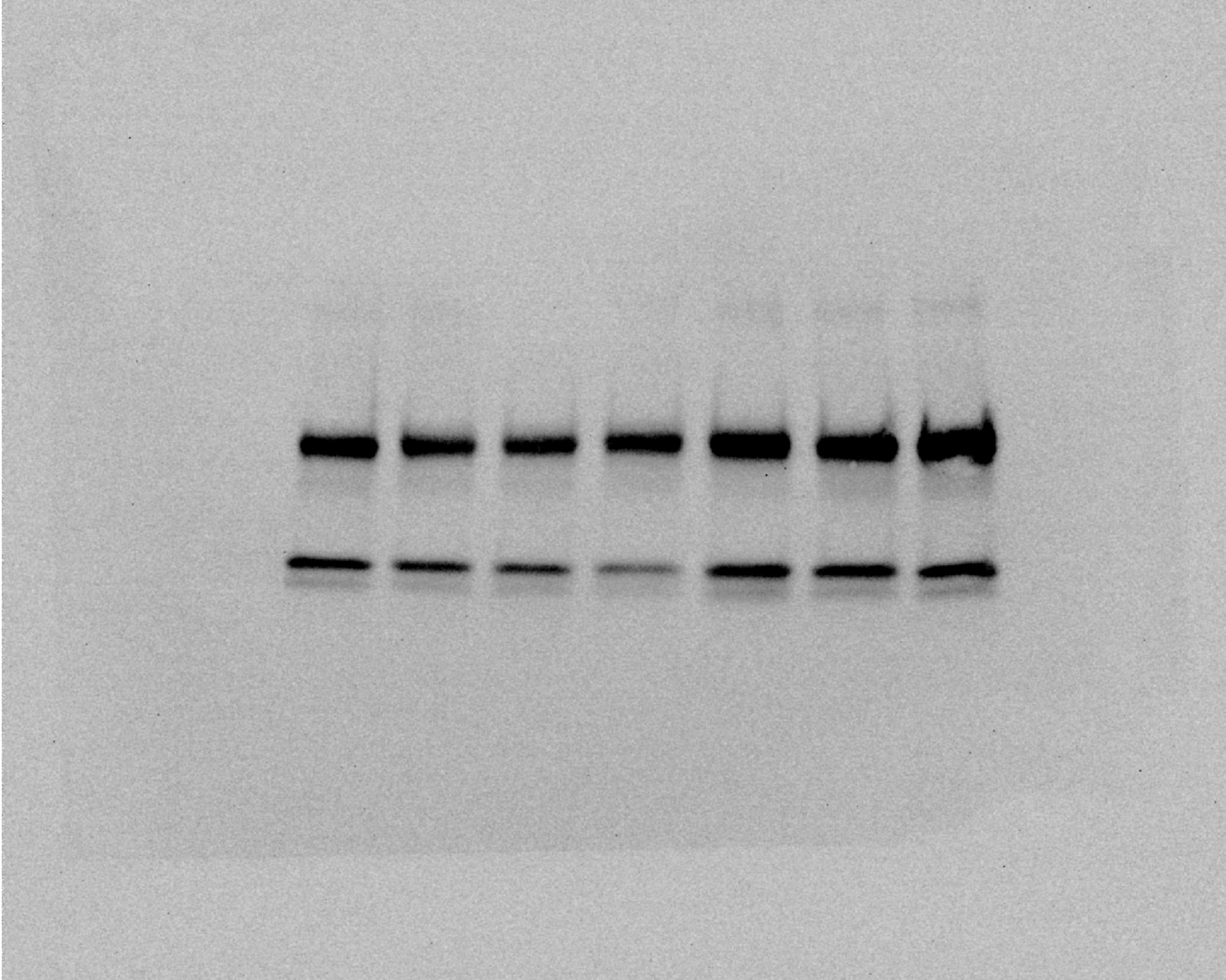

Supplement: Supplementary file 4 [file DataSheet2.zip › figure6-representative-blots/figure6-VDAC.tif]
