# Supplementary material for: Establishing a direct interaction between the 19,20-EDP analog SA-22 and SIRT3: impact on cardiac mitochondrial homeostasis
Source: Front Pharmacol. 2026 Jun 2;17:1805965. doi: 10.3389/fphar.2026.1805965 (PMC13268912; doi:10.3389/fphar.2026.1805965)
Supplement: Supplementary file 2 [file Table1.docx]

**(Supplementary Table 1.) Oxygen Consumption Rates [pmol/(s*Milli cells)]**

| **Respiratory**  **State** | **AEROBIC VEHICLE** | **AEROBIC NAM** | **AEROBIC 3-TYP** | **AEROBIC SA-22** | **HR VEHICLE 0 h** | **HR VEHICLE 3 h** | **HR VEHICLE 6 h** |
| --- | --- | --- | --- | --- | --- | --- | --- |
| **Basal** | 49.2 ± 1.5 | 51.3 ± 1.4 | 36.8 ± 4.3* | 56.8 ± 2.6 | 29.2 ± 3.1* | 29.7 ± 2.9* | 33.7 ± 3.7* |
| **PM_L_ (Digitonin)** | 27.3 ± 4.1 | 29.4 ± 1.2 | 22.7 ± 1.3 | 25.5 ± 1.6 | 18.9 ± 3.2 | 22.8 ± 3.6 | 20.6 ± 1.2 |
| **PM_p_ (ADP)** | 90.0 ± 16.2 | 84.5 ± 8.5 | 64.3 ± 8.6 | 96.1 ± 13 | 36.2 ± 7.4* | 36.5 ± 6.7* | 35.1 ± 5.0* |
| **PMc_P_ (Cyto. c)** | 92.9 ± 11.7 | 115.8 ± 8.3 | 94.5 ± 8.5 | 109.2 ± 6.9 | 52.9 ± 14.4 | 56.2 ± 12.1 | 39.2 ± 6.2 |
| **PMG_p_ (Glutamate)** | 105.9 ± 10.1 | 102.7 ± 7.2 | 82.1 ± 11.3 | 110.9 ± 7.3 | 65.5 ± 11.8 | 60.7 ± 10.9* | 63.4 ± 5.9* |
| **PMGS_P_ (Succinate)** | 184.4 ± 24.0 | 158.5 ± 11.9 | 121.7 ± 15.5 | 183.5 ± 8.0 | 82.9 ± 14.7* | 101.8 ± 20.8* | 73.9 ± 7.3* |
| **PMGS_E_ (FCCP)** | 236.7 ± 26.7 | 277.6 ± 19.7 | 182.2 ± 23.5 | 261.3 ± 23.2 | 78.7 ± 10.8* | 96.1 ± 14.7* | 106.8 ± 13.6* |
| **S_E_ (Rotenone)** | 5.0 ± 1.0 | 22.2 ± 7.9 | 30.3 ± 3.9 | 1.5 ± 0.4 | 38.3 ± 9.3 | 57.9 ± 12.0* | 32.7 ± 5.4 |
| **ROX (Antimycin A)** | 7.7 ± 1.5 | 5.4 ± 0.9 | 5.4 ± 1.3 | 6.0 ± 1.3 | 7.8 ± 4.2 | 8.8 ± 1.9 | 6.1 ± 1.7 |

| **Respiratory**  **State** | **HR SA-22**  **0 h** | **HR SA-22**  **3 h** | **HR SA-22**  **6 h** | **HR SA-22 +NAM**  **0 h** | **HR SA-22 +NAM**  **3 h** | **HR SA-22 +NAM**  **6 h** | **HR SA-22+ 3-TYP**  **0 h** | **HR SA-22+ 3-TYP**  **3 h** | **HR SA-22+ 3-TYP**  **6 h** |
| --- | --- | --- | --- | --- | --- | --- | --- | --- | --- |
| **Basal** | 52.8 ± 2.0 | 54.5 ± 2.9 | 45.8 ± 3.1 | 35.5 ± 1.3* | 24.7 ± 1.3* | 26.3 ± 1.4* | 35.1 ± 1.1* | 30.7 ± 2.5* | 28.0 ± 2.3* |
| **PM_L_ (Digitonin)** | 20.3 ± 3.0 | 21.0 ± 4.0 | 31.0 ± 2.9 | 19.6 ± 2.2 | 18.3 ± 1.6 | 21.2 ± 1.4 | 25.1 ± 0.8 | 23.7 ± 2.2 | 22.4 ± 2.9 |
| **PM_p_ (ADP)** | 62.5 ± 11.1 | 59.3 ± 12.2 | 83.6 ± 5.8 | 42.7 ± 7.8* | 25.8 ± 2.1* | 35.6 ± 5.9* | 71.2 ± 12.6 | 31.2 ± 3.2* | 26.0 ± 3.5* |
| **PMc_P_ (Cyto. c)** | 98.7 ± 27.2 | 70.2 ± 18.3 | 97.1 ± 15.3 | 62.2 ± 12.3 | 28.8 ± 3.6* | 38.8 ± 8.4 | 92.6 ± 14.2 | 32.8 ± 3.1* | 24.6 ± 3.5* |
| **PMG_p_ (Glutamate)** | 80.8 ± 16.6 | 84.0 ± 20.2 | 98.8 ± 10.7 | 70.2 ± 9.0 | 36.7 ± 2.8* | 43.6 ± 5.1* | 92.2 ± 7.4 | 39.6 ± 3.7* | 25.4 ± 3.5* |
| **PMGS_P_ (Succinate)** | 162.5 ± 36.4 | 153.9 ± 34.1 | 188.4 ± 13.3 | 130.8 ± 8.9 | 82.3 ± 7.8* | 84.5 ± 8.8* | 120.7 ± 6.1 | 111.4 ± 9.8 | 94.1 ± 15.2* |
| **PMGS_E_ (FCCP)** | 320.1 ± 45.4 | 275.8 ± 50.6 | 270.4 ± 42.5 | 174.3 ± 15.4 | 85.4 ± 9.8* | 100.1 ± 13.2* | 99.4 ± 5.8* | 123.5 ± 9.3* | 102.0 ± 13.5* |
| **S_E_ (Rotenone)** | 50.8 ± 29.2 | 8.9 ± 4.3 | 25.6 ± 17.5 | 75.5 ± 10.9* | 37.4 ± 8.6 | 50.3 ± 15.1 | 48.1 ± 6.7 | 71.7 ± 9.5* | 55.3 ± 10.1 |
| **ROX (Antimycin A)** | 7.5 ± 1.1 | 11.3 ± 3.0 | 12.3 ± 2.6 | 3.7 ± 0.8 | 3.4 ± 0.8 | 2.2 ± 0.6 | 2.2 ± 0.4 | 4.1 ± 0.7 | 3.4 ± 0.9 |
